# Intermittent fasting modulates the intestinal microbiota and improves obesity and host energy metabolism

**DOI:** 10.1038/s41522-023-00386-4

**Published:** 2023-04-07

**Authors:** Xiangwei Hu, Kai Xia, Minhui Dai, Xiaofeng Han, Peng Yuan, Jia Liu, Shiwei Liu, Fuhuai Jia, Jiayu Chen, Fangfang Jiang, Jieyao Yu, Huanming Yang, Jian Wang, Xun Xu, Xin Jin, Karsten Kristiansen, Liang Xiao, Wei Chen, Mo Han, Shenglin Duan

**Affiliations:** 1 BGI-Shenzhen, Shenzhen, 518083 China; 2 BGI College & Henan Institute of Medical and Pharmaceutical Sciences, Zhengzhou University, Zhengzhou, 450001 China; 3 Beijing Key Laboratory of the Innovative Development of Functional Staple and Nutritional Intervention for Chronic Diseases, China National Research Institute of Food and Fermentation Industries Co., Ltd, Beijing, 100015 China; 4 Department of Clinical Nutrition, Xiangya Hospital of Central South University, Changsha, 410008 China; 5 Ningbo Yufangtang Biological Technology Co., Ltd, Ningbo, 315012 China; 6 China National GeneBank, BGI-Shenzhen, Shenzhen, 518120 China; 7 Institute of Metagenomics, Qingdao-Europe Advance Institute for Life Sciences, BGI-Qingdao, 266555 Qingdao, China; 8 James D. Watson Institute of Genome Sciences, Hangzhou, 310058 China; 9 Guangdong Provincial Key Laboratory of Genome Read and Write, BGI-Shenzhen, Shenzhen, 518120 China; 10 Laboratory of Genomics and Molecular Biomedicine, Department of Biology, University of Copenhagen, DK-2100 Copenhagen, Denmark; 11 Shenzhen Engineering Laboratory of Detection and Intervention of Human Intestinal Microbiome, BGI-Shenzhen, Shenzhen, 518083 China; 12 Department of Clinical Nutrition, Peking Union Medical College Hospital, Chinese Academy of Medical Sciences & Peking Union Medical College, Beijing, 100730 China

**Keywords:** Microbiome, Metagenomics

## Abstract

Intermittent fasting (IF) is a promising paradigm for weight loss which has been shown to modulate the gut microbiota based on 16S rRNA gene amplicon sequencing. Here, 72 Chinese volunteers with a wide range of body mass index (BMI) participated in a three-week IF program during which an average loss of 3.67 kg body weight accompanied with improved clinical parameters was observed irrespective of initial anthropometric and gut microbiota status. Fecal samples were collected before and after the intervention and subjected to shotgun metagenomic sequencing. *De novo* assembly yielded 2934 metagenome-assembled genomes (MAGs). Profiling revealed significant enrichment of *Parabacteroides distasonis* and *Bacteroides thetaiotaomicron* after the intervention, with inverse correlations between their relative abundances and parameters related to obesity and atherosclerotic cardiovascular diseases (ASCVD). MAGs enriched after the intervention showed high richness and diversity of carbohydrate-active enzymes, with an increased relative abundances of genes related to succinate production and glutamate fermentation.

## Introduction

With the economic development and spread of modern Western diets and lifestyles, obesity has become a worldwide problem^1^. Compared to 1980, the obese population doubled in 2015, including 107.7 million children and 603.7 million adults. Moreover, more than 60% of 4 million annual global deaths have been related to obesity^2^. As a chronic metabolic disorder, obesity is known to be a predisposing factor for type 2 diabetes^3,4^, cardiovascular disease^4^, and several types of cancer^5^. To treat obesity and prevent associated diseases, different types of interventions have been applied and studied, including surgery, medication, exercise, and fasting.

As one strategy of fasting, intermittent fasting (IF) has shown consistent performance in relation to weight loss and relevant clinical improvements, attracting public attention in recent years^6^. A randomized controlled trial using a parallel-group design involving 115 obese women showed an average weight loss of 4 kg after a three-month IF intervention, whereas the average weight loss with traditional energy restriction by contrast was 2.4 kg. Moreover, the reduction in insulin resistance after IF (0.34 units in average) was greater compared to traditional fasting programs (0.20 units in average)^7^. Other studies also reported that the performance of IF intervention was better than that of physical training programs^8,9^. According to these results, IF holds promises as an option to counteract obesity. However, as most studies have been performed in obese western populations, it has not been well addressed whether IF intervention is beneficial for East-Asian populations or individuals with normal body weight and body fat.

The gut microbiota plays a significant role in obesity and associated diseases^10,11^. Changes in the composition and function potential of the gut microbiota can either prevent or promote obesity by stimulating the absorption of nutrients and regulating host metabolism^12–15^. Therefore, remodeling of the gut microbiota becomes an attractive strategy for the prevention and treatment of obesity^16,17^. Interplay between the gut microbiota and dietary habits has also been studied. Type and time of food intake were reported to modify the diurnal rhythm of the gut microbiota^18–20^. A previous study in mice also suggested that alterations in the gut microbiota in response to every-other-day fasting were associated with browning of white adipose tissue and a decrease in blood pressure^21,22^. However, IF-induced change in the human gut microbiota, especially at the species level, is not well characterized, and details on how this change may promote weight loss remain unclear.

In this study, we performed a three-week IF intervention in 72 Chinese adult participants with different body weights, ranging from regular to obese, and revealed pronounced improvements in multiple clinical parameters, including body weight, body mass index (BMI), and the atherosclerosis index (AI). IF-induced changes in the taxonomic composition and functional potential of the gut microbiota, and correlation between these changes and the clinical improvements were investigated based on a shotgun metagenomic sequencing approach. The results suggested that a short-term 5:2 IF intervention induced significant changes in the composition and functional potential of the gut microbiota in a baseline independent manner, associated with an increase in the relative abundance of species considered beneficial, including *Parabacteroides distasonis* and *Bacteroides thetaiotaomicron*, which may confer weight loss and improvements in host metabolism through the synthesis, and secretion of beneficial metabolites.

## Results

### Weight loss and clinical improvements after IF intervention

**Fig. 1:**
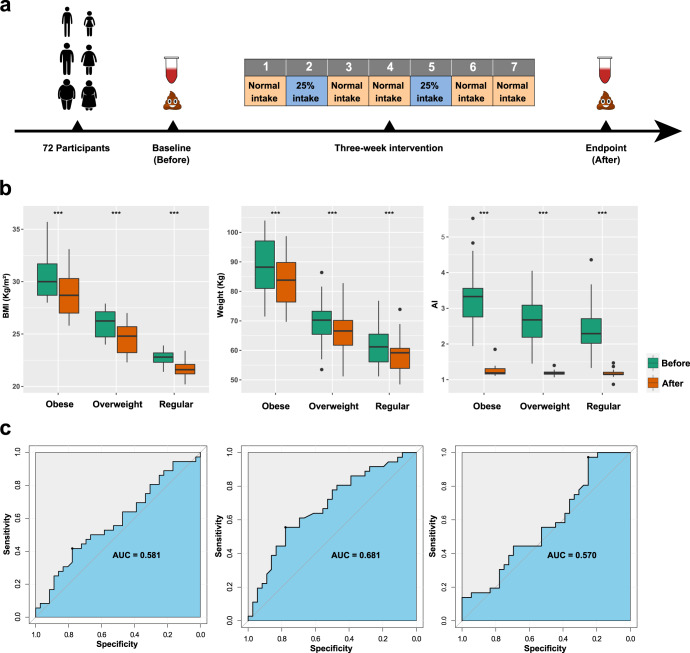
Study design and clinical improvements after the three-week IF intervention. **a** Schematic diagram of this study. For the five non-fasting days per week, meal replacement powders were arranged as a staple food for dinner, and participants were instructed to eat *ad libitum* (labeled as “Normal intake”). For the two discontinuous fasting days, the energy intake was set on 500–600 kCal (about 25% of participants’ normal intake), provided by meal replacement powders and protein sticks. **b** Body mass index (BMI), weight, and atherosclerosis index (AI) showed significant improvement (*** represents adjusted *p* < 0.001, paired Wilcoxon test) after the intervention. The improvements were uniform among participants with different levels of BMI. Bounds of boxes represent the first quartiles (Q1) and the third quartiles (Q3) respectively, center lines represent the median values, and the whiskers are ranged from Q1–1.5 × (Q3–Q1) to Q3 + 1.5 × (Q3–Q1). **c** Receiver operating characteristic (ROC) curve of random forest classifiers based on the relative abundances of metagenome-assembled genomes (MAGs) before the intervention. Area under the curve (AUC) = 0.570, 0.681, and 0.582, respectively.

We performed a three-week intervention study on 72 adult Chinese volunteers subjected to a 5:2 program as detailed in Fig. 1a. The volunteers comprised individuals ranging from regular to obese (Supplementary Table 1). Seventeen clinical parameters were determined both before and after the intervention for all participants. We observed significant improvements in obesity related parameters, including BMI, weight, and AI (Fig. 1b, Supplementary Fig. 1 and Supplementary Table 1). Body weight decreased on average 3.67 kg (adjusted *p* < 0.0001), BMI decreased on average 1.32 (adjusted *p* < 0.0001), whereas AI decreased on average 1.53 (adjusted *p* < 0.0001). These changes testified to the efficiency of the 5:2 program applied in this study.

The results further demonstrated that the clinical improvements induced by IF intervention were not restricted to the obese participants but were also observed in individuals with normal BMI. For example, participants with different levels of BMI lost on average 2.82 kg (regular, *p* < 0.0001), 3.84 kg (overweight, *p* < 0.0001), and 4.82 kg (obese, *p* = 0.0003) of body weight after the intervention, and AI also decreased 1.23 (*p* < 0.0001), 1.50 (*p* < 0.0001), and 2.10 (*p* < 0.0001) respectively. For most of the parameters that were measured, the trends in IF-induced clinical changes were consistently observed across obese (*n* = 17), overweight (*n* = 26), and regular (*n* = 29) individuals (Fig. 1b and Supplementary Fig. 1). Of note, random forest classifiers based on the taxonomic composition of gut microbiota before the intervention showed poor performance in predicting whether the improvement in BMI, weight, or AI of a specific participant was higher or lower than the median, (Fig. 1c), suggesting that major responses to the IF intervention did not depend on the baseline taxonomic composition of the gut microbiota as observed in a study investigating general caloric restriction^23^.

### IF intervention causes significant changes in the taxonomic composition of the gut microbiota

To characterize the effects of the IF intervention on the gut microbiota, participants were asked to donate fecal samples before and after the intervention. Seventy-two pairs of matched samples were obtained, and shotgun metagenomic sequencing was then performed.

**Fig. 2:**
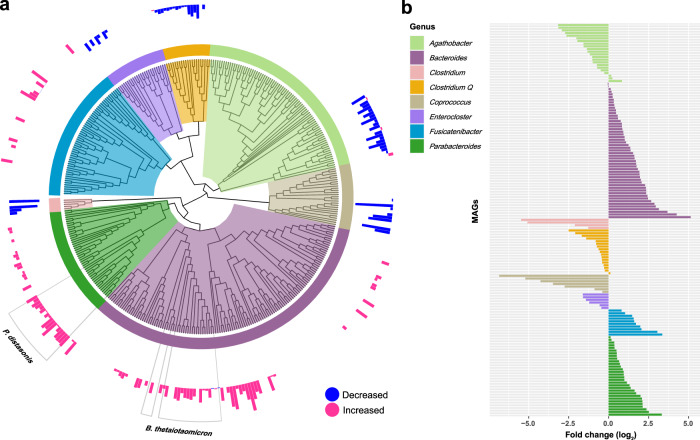
Changes in the taxonomic composition of the gut microbiota after IF intervention. **a** Phylogenetic tree of genera in which only metagenome-assembled genomes (MAGs) either enriched before or after the intervention are included. The bar-plot circle outside represents the fold change of the MAGs. The magenta bar indicates significant enrichment after the intervention, whereas the blue bar indicates a decrease in abundance after the intervention (adjusted *p* < 0.05, paired Wilcoxon test). The tree was constructed by PhyloPhlAn (v3.0.51) and visualized by *ggtree* (v5.6.2). **b** Fold change of the MAGs enriched either before or after the intervention. The MAGs with a fold change less than zero were enriched before the IF intervention, and those with values above zero were enriched after.

In total, 2934 MAGs, including 241 known genera and 538 known species, were recovered by *de novo* assembling and binning. A total of 201 MAGs were found to differ in abundances comparing samples taken before and after the intervention, with 147 being annotated as known species of *Parabacteroides* (30 MAGs), *Bacteroides* (51 MAGs), *Agathobacter* (22 MAGs), *Fusicatenibacter* (10 MAGs), *Clostridium Q* (17 MAGs), *Clostridium* (4 MAGs), *Coprococcus* (7 MAGs), and *Enterocloster* (6 MAGs) (Supplementary Table 2). In particular, a vast majority of the MAGs enriched after the intervention were annotated as *Parabacteroides* spp., *Bacteroides* spp., and *Fusicatenibacter* spp., whereas MAGs exhibiting a decrease in abundance after the intervention were annotated as *Agathobacter* spp., *Clostridium* spp., *Clostridium Q* spp., *Coprococcus* spp., and *Enterocloster* spp. (Fig. 2). MAGs annotated as *Parabacteroides merdae*, *Parabacteroides distasonis*, *Fusicatenibacter saccharivorans*, *Bacteroides uniformis*, *Bacteroides thetaiotaomicron*, and *Bacteroides cellulosilyticus* showed on average relatively high positive fold-changes, whereas those annotated as *Coprococcus* sp900066115 and *Agathobacter rectalis* on average showed relatively high negative fold-changes (Supplementary Fig. 2).

Notably, approximately one-third of the MAGs annotated as *Parabacteroides* and *Bacteroides* while exhibiting an increase in abundance after the IF intervention, were assigned to *P. distasonis* and *B. thetaiotaomicron*; species that previously have been shown to exert beneficial effects on energy metabolism and obesity development^23–25^.

### Species enriched after IF intervention harbor abundant and diverse carbohydrate-active enzymes (CAZymes)

**Fig. 3:**
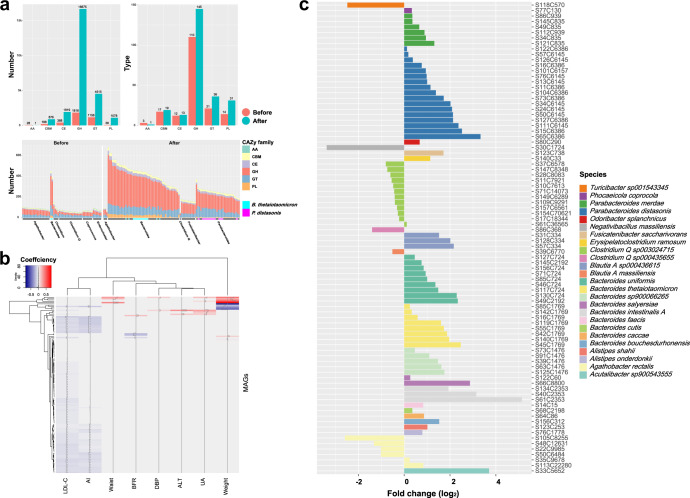
Richness and diversity of carbohydrate-active enzymes in MAGs enriched before and after the intervention. **a** Comparison in counts of carbohydrate-active enzyme subfamilies and copy number of carbohydrate-active enzymes (CAZymes) encoding genes observed in the metagenome-assembled genomes (MAGs) enriched before and after the IF intervention. **b** Heatmap of associations between the relative abundances of MAGs and clinical parameters of participants. Red represents a positive correlation, whereas blue represents a negative correlation. *AI* atherosclerosis index; *ALT* alanine aminotransferase; *BFR* body fat ratio; *DBP* diastolic blood pressure; *LDL-C* low-density lipoprotein cholesterol; *UA* uric acid. **c** Enrichment of MAGs negatively correlated with AI. The MAGs with a fold change less than zero were enriched before the IF intervention, and those with values above zero were enriched after.

We investigated the diversity of carbohydrate-active enzymes in the 147 MAGs with taxonomic annotation at the species level and differing in abundance between samples taken before and after the intervention. More types and higher copy numbers of carbohydrate-active enzyme coding genes were found in MAGs exhibiting higher abundance after the IF intervention. Most of these genes were annotated as glycoside hydrolases (GHs) and glycosyltransferases (GTs), suggesting that the ability of the gut microbiota to use diverse carbon sources was enhanced. (Fig. 3a) Notably, *Bacteroides* spp. mainly contributed to the richness of polysaccharide lyases (PLs), whereas very few PL-coding genes were identified in MAGs of other genera, especially those present in higher abundance before the intervention.

### IF-induced changes in the gut microbiota correlate with clinical parameters of host metabolism and obesity

To further evaluate whether the changes in the gut microbiota were correlated to blood biochemical parameters in participants and clinical improvements after the intervention, we determined the correlation between the relative abundances of MAGs and clinical measurements of the hosts. In total, 136 MAGs were found to be significantly correlated with at least one of the clinical parameters (Fig. 3b and Supplementary Table 3). In particular, 86 MAGs of 25 species, including 8 MAGs of *B. thetaiotaomicron* and 17 MAGs of *P. distasonis*, were negatively associated with AI, and 84 of them were also negatively associated with serum low-density lipoprotein cholesterol (LDL-C).

Notably, 67 of the 86 MAGs which negatively correlated with AI, including all the 25 MAGs annotated as *P. distasonis* and *B. thetaiotaomicron*, were enriched after the IF intervention, whereas most of the MAGs that exhibited higher relative abundances before the intervention were assigned to *Clostridium Q* sp003024715 and *Agathobacter rectalis* (Fig. 3c).

### IF intervention increases the potential for succinate synthesis by the gut microbiota

In addition to changes in the taxonomic composition, we also determined changes in the functional potential of the gut microbiota. Clean reads were mapped to the published gut microbial gene catalog^26^ to calculate the abundances of functionally annotated genes.

**Fig. 4:**
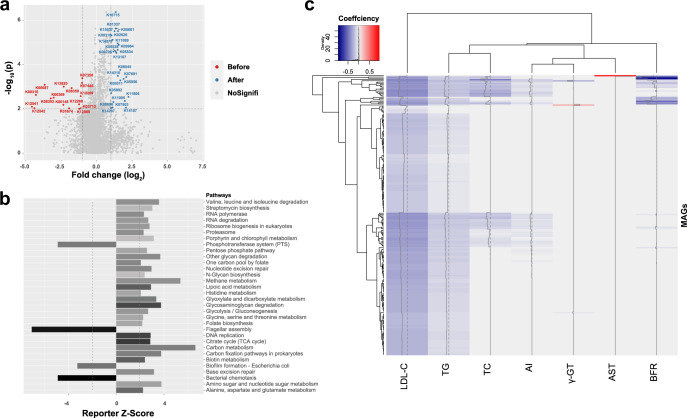
IF intervention changes the functional potential of the gut microbiota. **a** In the volcano plot, each dot represents a KEGG Orthologues (KO) and is distinguished by color. KOs enriched before the experiment are shown in red, and KOs enriched after the intervention are shown in blue. **b** The *X* axis represents the reporter *Z* score, and the *y* axis represents the functional pathways. The pathways with Z-scores less than zero were enriched before intervention, and those with values above zero were enriched after the intervention. The darkness of the bars indicates the completeness of each pathway. **c** Heatmap of associations between the relative abundances of KOs and clinical parameters. Red represents positive correlation, whereas blue represents negative correlation. *AI* atherosclerosis index, *AST* aspartate aminotransferase; *BFR* body fat ratio, *DBP* diastolic blood pressure, *γ-GT* gamma-glutamyl transferase, *LDL-C* low-density lipoprotein cholesterol, *TC* total cholesterol, *TG* triglyceride.

Using a threshold of adjusted *p* < 0.01 and |fold-change|> 1, 25 KEGG Orthologues (KOs) were significantly enriched after the IF intervention, whereas the relative abundance of 16 KOs decreased (Fig. 4a). We further calculated the reporter *Z* score to determine the enrichment of functional pathways. The results highlighted that carbon metabolism, the citrate cycle, and the pentose phosphate pathways were significantly enhanced after the intervention, which possibly would lead to higher carbohydrate conversion and increased short-chain fatty acid (SCFA) production by the gut microbiota (Fig. 4b).

We further analyzed the correlation between the relative abundances of KOs and the clinical parameters. A total of 220 KOs were identified to be correlated with at least one clinical parameter (Fig. 4c and Supplementary Table 4). High uniformity was observed between KOs negatively correlated with serum LDL-C and serum triglyceride (TG), as well as those negatively correlated to serum total cholesterol (TC), AI, and body fat. Notably, the relative abundances of three KOs, K00844 (hexokinase), K06859 (glucose-6-phosphate isomerase), and K15037 (biotin carboxyl carrier protein), which play roles in the synthesis of succinate, (Supplementary Fig. 3), K03146 (cysteine-dependent adenosine diphosphate thiazole synthase), which is related to thiamine synthesis, (Supplementary Fig. 4), and K05681 (ATP-binding cassette, subfamily G), which is involved in bile secretion, (Supplementary Fig. 5) were significantly increased after the intervention, but negatively correlated with the five parameters closely related to hyperlipidemia and atherosclerotic cardiovascular disease (ASCVD). Finally, K06045 (squalene-hopene/tetraprenyl-beta-curcumene cyclase) involved in the synthesis of hopene was positively associated with serum gamma-glutamyl transferase (γ-GT), whereas the uncharacterized K08087 was positively correlated with serum aspartate transaminase (AST).

## Discussion

Although both time-restricted feeding (TRF) and alternative day IF (ADF) have been reported to be effective means for weight loss in elderly and obese individuals^27,28^, there are no detailed information as to whether these programs, or any other IF programs, also would elicit beneficial clinical changes in individuals with normal weight and body fat. In this study, 72 volunteers, including healthy regular weight (18.5 kg/m^2^ ≤ BMI < 24.0 kg/m^2^) to overweight (24.0 kg/m^2^ ≤ BMI < 28.0 kg/m^2^) and obese (BMI ≥ 28 kg/m^2^), lost 3.67 kg on average of weight by adhering to a 5:2 IF program for three weeks, without any engagement in additional physical training or diet control in the non-fasting days. Clinical parameters were also improved, especially those related to obesity, hyperlipidemia, and ASCVD. Moreover, in contrast to the conclusions obtained from a weight loss study using general caloric restriction^23^, the baseline gut microbiota did not affect the performance of the IF program applied in this study. The results of this study thus suggest that IF intervention may be a promising strategy for weight loss in populations within a wide range of BMI and irrespective of the composition of the gut microbiota.

A previous mouse study reported that the gut microbiota may contribute to every-other-day fasting-induced white adipose tissue browning, and reported that the relative abundances of *Bacteroides* and *Parabacteroides* decreased simultaneously^21^. However, it remains unclear whether a similar correlation also exists in humans and for other IF programs. Other mouse studies have investigated the changes in gut microbiota induced by different dietary fasting programs but rarely reported on significant change in the relative abundance of *Bacteroides* or *Parabacteroides*^29^. Relatively few human studies have been conducted to assess IF-induced changes in the gut microbiota and the relationship with weight loss and host metabolism, and even for these few studies, only the 16S rRNA gene amplicon sequencing approach was applied, hampering analyses at the species level^29,30^. These studies reported that ADF and Ramadan would lead to an increase in the relative abundance of Bacteroidaceae and *Bacteroides* in multiple sclerosis patients and healthy adults, respectively^31,32^, whereas other reported that IF-induced changes in taxonomic composition of gut microbiota were not consistent in published articles. In this study, we observed that the relative abundances of multiple *Bacteroides* and *Parabacteroides* species, especially the reported beneficial bacteria *P. distasonis* and *B. thetaiotaomicron*, were significantly increased after the IF intervention. This change is different from what has been reported in previous mouse studies, underscoring the differences between the mouse and the human gut microbiota, suggesting that the responses to IF interventions and the associated molecular mechanisms may differ. Our analyses showed a significant negative correlation between the relative abundances of the IF-enriched *B. thetaiotaomicron* and *P. distasonis*, and serum LDL-C and AI, suggesting that these bacteria may play a role in the beneficial effect of the IF intervention. Of note, it has been reported that the relative abundances of *Bacteroides* spp. and *P. distasonis* are both decreased in the ASCVD patients^33^.

To further examine the association between IF, IF-induced change in the gut microbiota, and clinical improvements in the participants, changes in the functional potential of the gut microbiota were investigated. By analyzing the richness and diversity of carbohydrate-active enzymes, we found that the majority of the species enriched after the IF intervention, mostly *Bacteroides* spp. and *Parabacteroides* spp., showed a remarkably high richness and diversity of genes encoding GHs, GTs, and PLs. The robust increase in the abundance of microbes capable of digesting diverse carbohydrates and glycoproteins may be a consequence of the intermittent shortage of carbon sources induced by the IF intervention. Notably, *Bacteroides* spp. were found to carry high counts of PL encoding genes, whereas very few genes of this family were observed in other species investigated, suggesting a unique role for *Bacteroides* spp. in the microbial response to dietary fasting interventions.

According to previous reports, the enrichment of *P. distasonis* may alleviate obesity and metabolic dysfunctions in mice via the production of succinate and the activation of intestinal gluconeogenesis^25^, whereas the enrichment of *B. thetaiotaomicron* may increase fermentation of glutamate to gamma-aminobutyric acid (GABA), and therefore reduce plasma glutamate concentrations^24,34^. Consistently, in this study, we observed increases in the abundance of KOs related to succinate synthesis and glutamate metabolism after the intervention, as well as a negative correlation between these KOs and clinical parameters of obesity, hyperlipidemia, and ASCVD. The simultaneous increase in the relative abundances of the species and the functional genes supports a hypothesis that IF intervention may elicit an enrichment of CAZyme-rich gut bacteria, including *P. distasonis* and *B. thetaiotaomicron* which contribute to alleviate obesity and complications through production of succinate and GABA.

Since the main objective of this study was to investigate how intermittent fasting may affect the gut microbiota and host physiological parameters, and the potential links between these factors, a single-group design was applied to maximize the number of individuals who adhered to the IF dietary pattern, which would improve statistical power of relevant analyses under the limitations associated with the total number of participants. However, the absence of a control group made it impossible to perform comparison between IF and a normal dietary pattern in this study. Although the pronounced and statistically significant clinical improvement observed after the intervention suggests that the intervention was sufficient, uncontrolled covariates might also contribute. Further large-scale randomized clinical trials are required to validate our findings and to better distinguish effects induced by the IF intervention per se and the changes in behavior during the program which were not noticed by the participants themselves.

Although further in vitro and in vivo studies are necessary to elucidate the relationship between IF, gut microbiota, and host metabolism, the results of this study suggest that IF-induced changes in gut microbiota can contribute to weight loss and other associated clinical improvements rather than being a “side effect” of the improvements directly caused by the IF intervention. Finally, the genetic and functional features of other bacteria which were also enriched after the IF intervention, especially *Fusicatenibacter saccharivorans*, have not yet been well characterized. The possible role of these microorganisms warrants further studies.

Recent review articles have summarized human trials concerning IF and its effects on the gut microbiota^29,30^. As a significant advantage of the current study, deep metagenomic sequencing and *de novo* assembly, rather than 16S rRNA gene amplicon sequencing, were performed providing information regarding the gut microbiota at the level of species or even strains, as well as information regarding the functional potential. Association analyses based on the detailed metagenomic data further revealed correlations between clinical parameters and specific gut microorganisms. In addition, the relatively large number of participants compared to previous human studies involving less^35^ participants, enabled analyses of participants with a large range of BMI, and suggested a uniform trend of clinical improvements irrespective of BMI.

However, several limitations of this study should also be addressed. As mentioned above, a control group was not included in the study, and strict adherence to the usual *ad libitum* diets was not supervised by medical personnel. Although the participants were instructed to document all food intake, and asked to follow their normal routines during the three-week intervention, uncontrolled covariates could also contribute to the observed changes. Besides, although it has been reported that the atherogenic index of plasma (AIP) takes TG into account and may provide better performance in prediction of potential cardiovascular events^35,36^, it is log-transformed and might induce problems in specific regression analysis. Thus, we used AI, which is still used by many of the clinical doctors due to its advantages in simplicity, but not AIP in this study. Furthermore, gut transit time was not measured in this study. Since previous studies have reported that the gut transit time influences the composition of the gut microbiota^37–39^, interactions between IF and the gut microbiota may be modulated by this factor. Finally, experiments to investigate and validate the mechanisms linking IF, gut microbiota, and host metabolism were not performed in this study.

## Methods

### Design of the study and enrollment of participants

The intervention was conducted at Xiangya Hospital, Hunan, China. The design of the study and protocols were approved by the Medical Ethics Committee of Xiangya Hospital, Central South University, and the Institutional Review Board of BGI. Written informed consent was obtained from each participant. The study was conducted in accordance with the approved guidelines and regulations. The inclusion criteria were as follows: (i) 18–55 years old and (ii) 18 kg/m^2^ < BMI < 30.0 kg/m^2^. The exclusion criteria were (i) antibiotic therapy during the last 4 weeks; (ii) a diagnosis of hypertension, diabetes, or other metabolic diseases; (iii) pregnancy, gastrointestinal abnormalities or eating disorders, history of gastrointestinal surgery or systemic diseases; and (iv) use of corticosteroid drugs, β-receptor blockers, or other drugs that might affect the findings. Ninety-four volunteers signed consent forms and were evaluated according to the criteria of the study. Eighty-one of them fulfilled the criteria and participated in the whole trial. Seventy-two of the participants donated all required samples.

### Intervention, general body information collection, and blood biochemical assay

The IF intervention was conducted following the 5:2 program for three weeks with some minor adjustment. For the two discontinuous fasting days, the energy intake was set on 500–600 kCal, provided by meal replacement powders and protein sticks. Participants were asked to stay in a sanatorium with no access to additional foods in the fasting days. For the other five days per week, meal replacement powders were arranged as a staple food for dinner, and normal diets were provided so participants might eat *ad libitum*. Participants were asked to take photographs of every meal in the non-fasting days and send these pictures to nurses and doctors at Xiangya Hospital each day for evaluation of the compliance. In particular, if any of the participants did not send the photographs, or consumed obviously too less/much, they would be noticed for the first time, and would be marked as “to be excluded in analyses” at the second time. Participants were asked to adhere to their usual habits in exercises and social activities during the whole program, and acknowledged to follow all rules above by signing the informed consent forms. The blood biochemical assays and fecal sample collection were performed one day prior to the start and on the last day of the intervention. For the blood biochemical assays and physical examination, 17 clinical parameters were measured, with AI being calculated as (TC - HDL-C)/HDL-C. (Supplementary Table 1)

### Fecal DNA extraction and shotgun metagenomic sequencing

A total of 175 fecal samples from participants were collected before and after the IF intervention using the MGIEasy Stool Sample Collection Kit (Item No. 1000003702, MGI Tech Co., Ltd., China) based on the reagent assessed previously^40^. DNA was extracted with MagPure Fast Stool DNA KF Kit B (MD5115, Magen Biotechnology Co., Ltd, China) and quantified in China National GeneBank (CNGB) following the manufacturer’s instructions^41^. Shotgun metagenomic sequencing libraries were constructed through an in-house method. In particular, 200 ng DNA of each sample was sheared by Covaris S220 (Covaris LLC., USA) into fragments with approximate length of 300 bp without size selection, and downstream reactions were performed using MGIEasy Universal DNA Library Prep Set (Item No. 1000006985, MGI Tech Co., Ltd., China). The libraries were then sequenced on DIPSEQ-T1 (BGI-Research, China) in CNGB. In total, 25.96 ± 12.17 (mean ± s.d.) Gbp of PE150 raw data per sample were obtained. Quality control of sequencing data was performed using the module of the internally developed cOMG toolkit based on the algorithm of overall accuracy, and generated 25.12 ± 11.84 (mean ± s.d.) Gbp of clean reads per sample^42^. Metagenomic sequencing data obtained from all 175 collected fecal samples were used for *de novo* assembling and binning, whereas only 144 records of the 72 participants who donated all required samples and information were included in other analyses.

### *De novo* metagenomic assembling and contig binning

Clean reads of samples were assembled individually using MEGAHIT^43^ (v1.1.3). VAMB^44^ (v3.0.1) was then used for metagenome binning with the option *--minfasta 10000 -e 400*. Bins of metagenomes were dereplicated by dRep^45^ (v3.2.0) with the option *dereplicate -sa 0.99 -nc 0.3 -p 6 -comp 80 -con 10* to obtain the nonredundant MAG catalog. Profiling was performed via Salmon using the *quant_bins* module of metaWRAP (v1.3.2) to calculate the abundances of MAGs in each sample.

### Taxonomic annotation of the MAGs

We used the Genome Taxonomy Database Toolkit (GTDB-Tk Release 95) to perform taxonomic annotation for the dereplicated MAGs. Bins with average nucleotide identity (ANI) < 95% compared to any genome of known species were considered unknown species-level genome bins (uSGBs).

### Phylogenetic analysis of the MAGs

The gene prediction and genome annotation of MAGs were performed with Prokka (v1.14.5) and the bins annotating module of metaWRAP (v1.3.2), respectively. The phylogenetic tree of the 2934 representative MAGs was further built by PhyloPhlAn (v3.0.51) and visualized using the *ggtree* package (v2.3.3.993) in R (v3.6.2).

### Analysis of the compositional changes in the microbiota and general statistics

Data from 144 pairwise fecal samples from 72 participants were included in the analysis. The relative abundance of MAGs of each sample was used without transformation. Permutational multivariate analysis of variance (PERMANOVA) was performed using the *adonis()* function in the *vegan* package (v2.5-7). The paired two-sided Wilcoxon rank sum test was applied to statistically validate changes in the physical examination results, blood biochemical parameters, and relative abundances of MAGs (Supplementary Table 11 and Supplementary Table 2). Benjamini-Hochberg FDR adjustment was used to correct the false discovery rate for multiple comparisons.

### Analysis and comparison of the microbial functional potentials

Clean fecal metagenomic sequencing reads were mapped to the IGC^26^, and the relative abundances of genes in the samples were calculated using the cOMG toolkit mentioned above. The original KO annotation of IGC was used to annotate the profiles. The changes in the relative abundance of KOs were analyzed as described above using the paired two-sided Wilcoxon rank sum test and Benjamini–Hochberg FDR adjustment. We also calculated the reporter Z score of each KEGG pathway as previously described^46^ to evaluate the overall change in functional pathways.

### Metagenome-wide association study

Associations between gut microorganisms and clinical parameters were analyzed by applying an ℓ1-penalized robust regression method^47^. The numeric results of the physical examination and blood biochemical parameters were used as the response variables, whereas the log_10_-transformed relative abundances of MAGs or KOs enriched either before or after the intervention served as the predictor variable matrix. The regression was performed in R (v4.1.2) using the *ILAMM* package (v1.0.0). The function *tfNcvxHuberReg()* was used with the option *intercept* = *T*, whereas other parameters and options were set by default.

## Supplementary information

Supplemental figures and tables

Supplemental codes

## Data Availability

Host-removed metagenomic sequencing data are available on CNGB Sequence Archive (CNSA, https://db.cngb.org/cnsa/) with accession number CNP0002844. Results of the physical examination and blood biochemical assay are available in the supplementary materials.

## Code availability

Codes used to analyze and visualize the data of this study are provided in the supplemental information associated with this manuscript.
