# Supplementary material for: Intermittent fasting modulates the intestinal microbiota and improves obesity and host energy metabolism
Source: NPJ Biofilms Microbiomes. 2023 Apr 7;9:19. doi: 10.1038/s41522-023-00386-4 (PMC10081985; doi:10.1038/s41522-023-00386-4)
Supplement: Supplementary file 1 — Supplemental figures and tables [file 41522_2023_386_MOESM1_ESM.pdf]

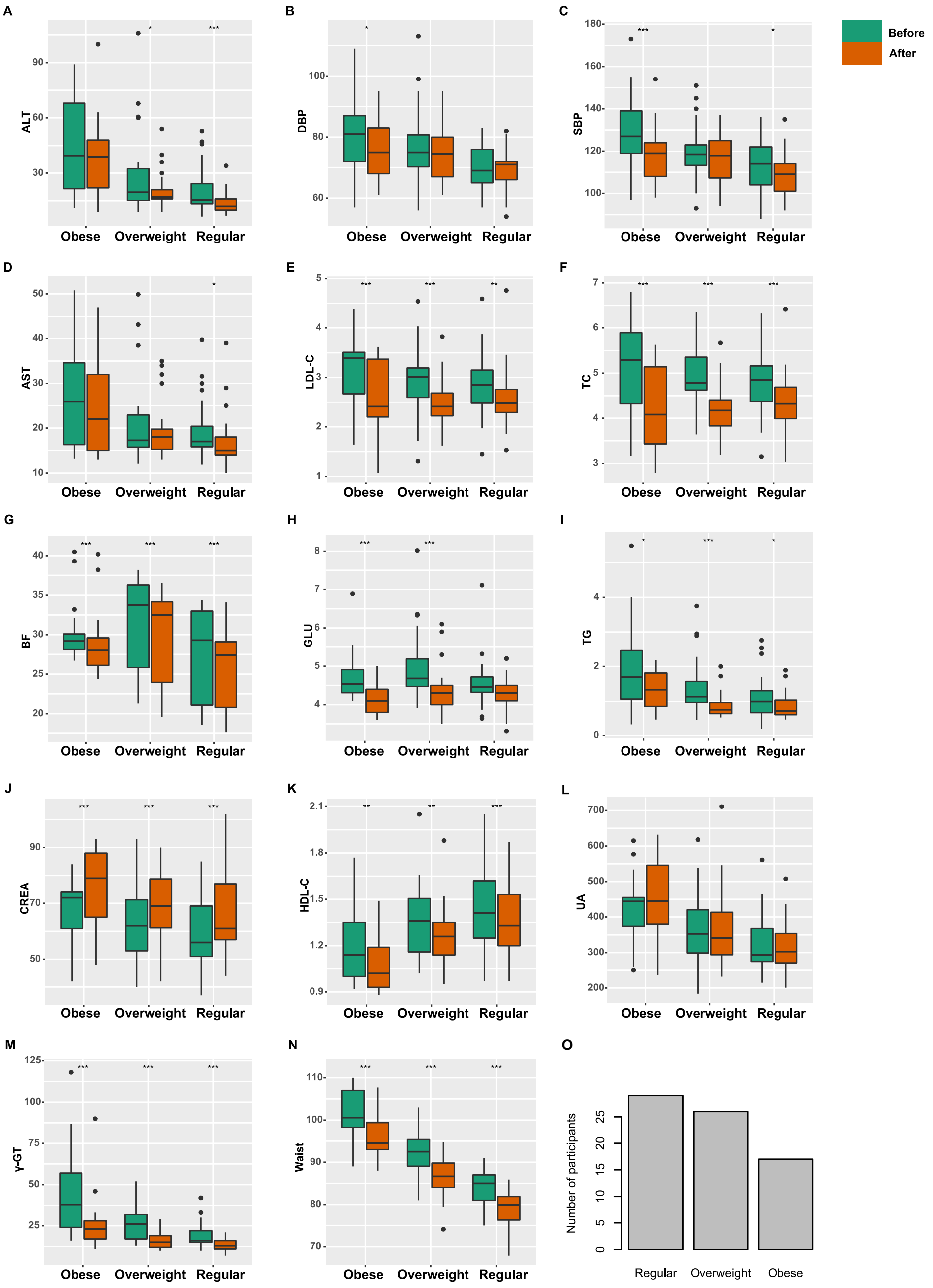

Supplementary Figure 1. Changes in clinical parameters after the three-weeks IF intervention. Most of the parameters showed significant improvement (*** represents adjusted $p < 0.001$, ** represents adjusted $p < 0.01$, * represents adjusted $p < 0.05$, respectively, paired Wilcoxon test) after the intervention. The improvements were mostly uniform among different levels of body mass index (BMI). (**A**) Atherosclerosis index (AI). (**B**) Diastolic blood pressure (DBP). (**C**) Systolic blood pressure. (**D**) Aspartate aminotransferase (AST). (**E**) Low-density lipoprotein cholesterol (LDL-C). (**F**) Total cholesterol (TC). (**G**) Body fat (BF). (**H**) Glucose (GLU). (**I**) triglyceride (TG). (**J**) Creatinine (CREA). (**K**) High-density lipoprotein cholesterol (LDL-H). (**L**) Uric acid (UA). (**M**) Gamma-glutamyl transferase (γ-GT). (**N**) Waist. (**O**) Distribution of the participants to the three BMI classes. Plots of BMI, weight, and atherosclerosis index (AI) are presented in the main Figure 1B and

therefore not included here. Bounds of boxes represent the first quartiles (Q1) and the third quartiles (Q3) respectively, centre lines represent the median values, and the whiskers are ranged from Q1 – 1.5 × (Q3 – Q1) to Q3 + 1.5 × (Q3 – Q1).

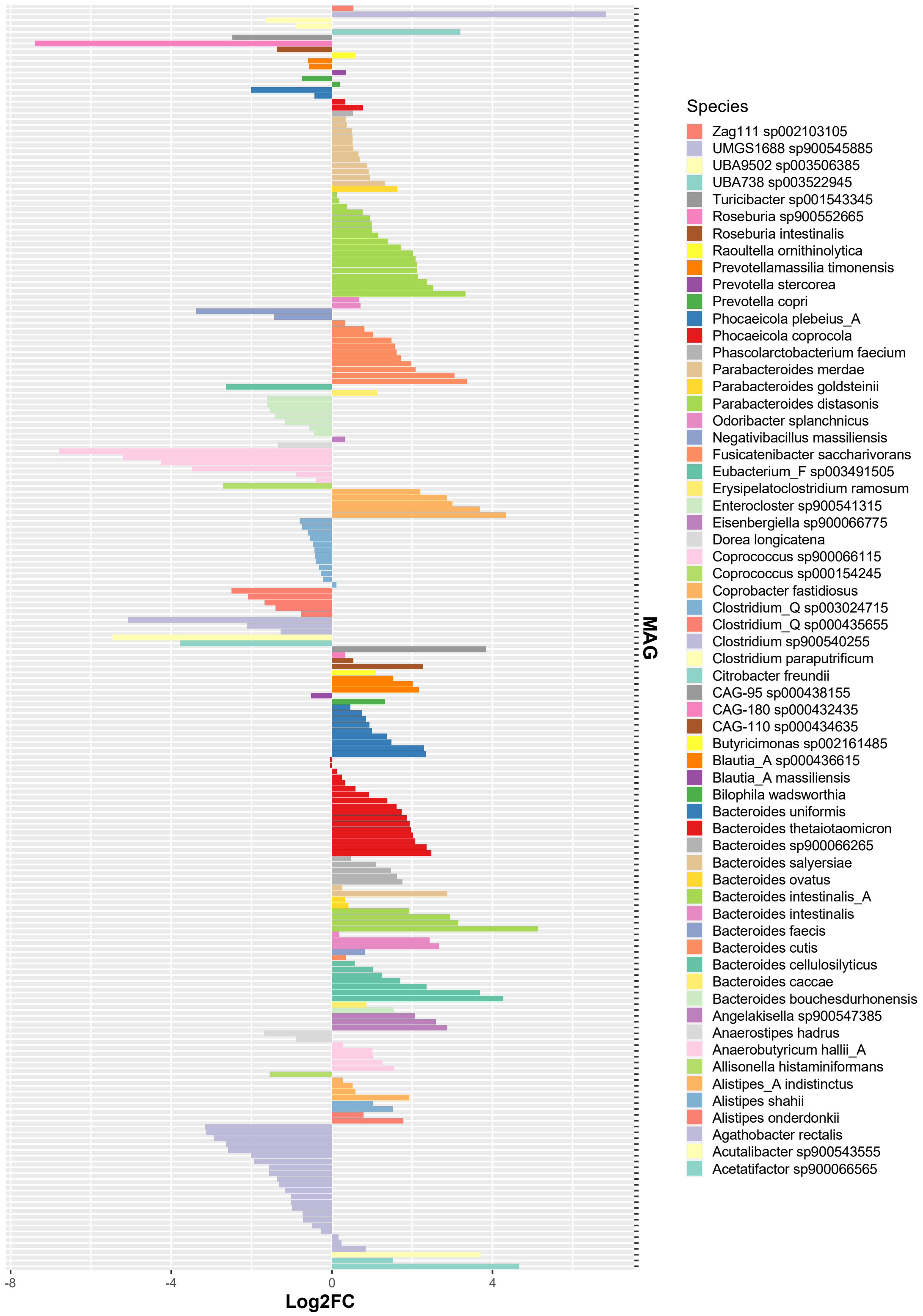

Supplementary Figure 2. Fold change of the MAGs enriched either before or after the intervention. The metagenome-assembled genomes (MAGs) with a fold-change less than zero were enriched before the IF intervention, and those with values above zero were enriched after. Bars of MAGs are colored by species or unknown

species-level genome bins (uSGBs). Some of the colors are used multiple times, therefore the barplot and the legend is arranged in identical order for convenience.

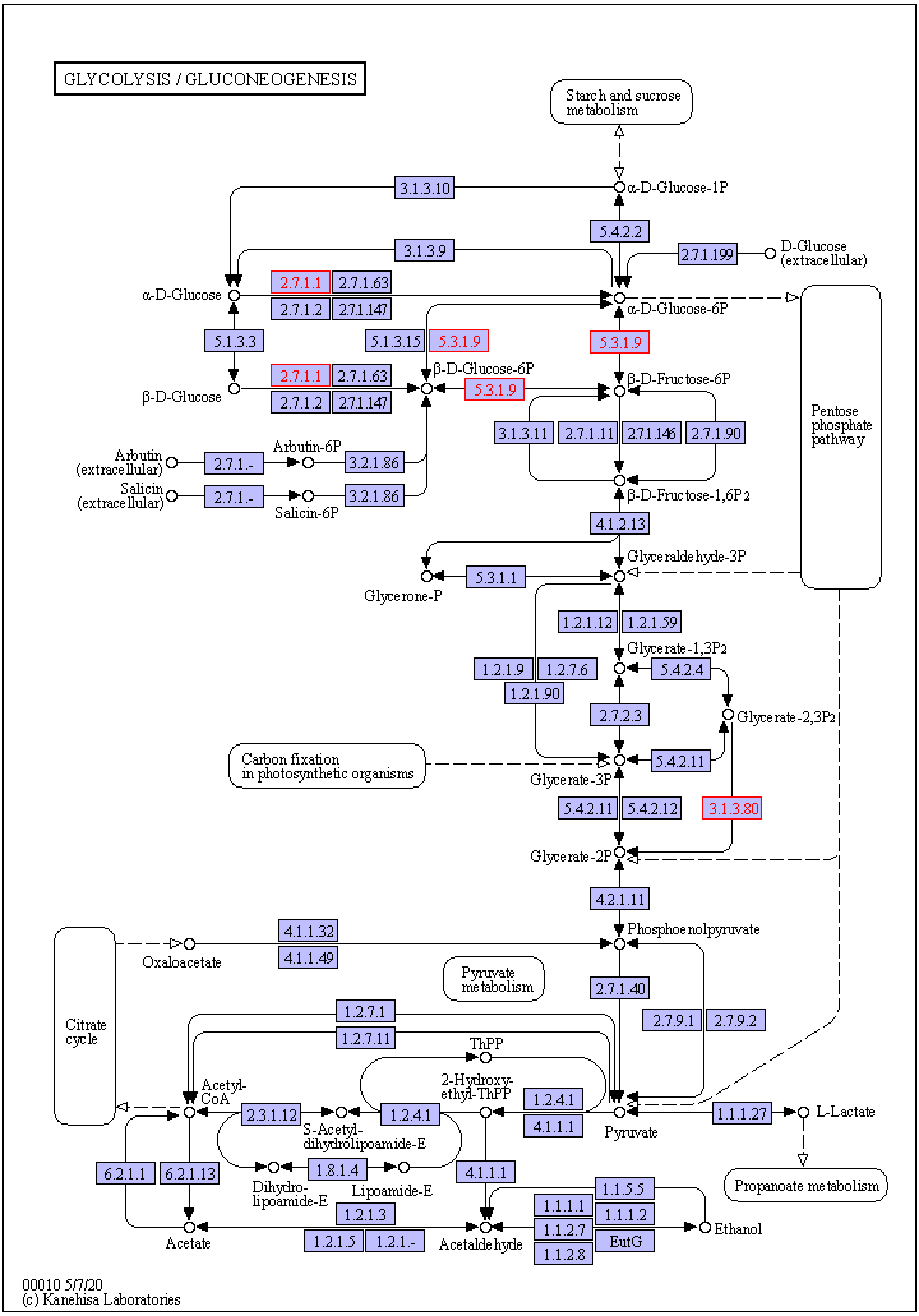

Supplementary Figure 3. Diagram of the carbon metabolism pathway (KEGG map01200). K00844, K06859, and K15037 were enriched after the IF intervention and labeled in red.

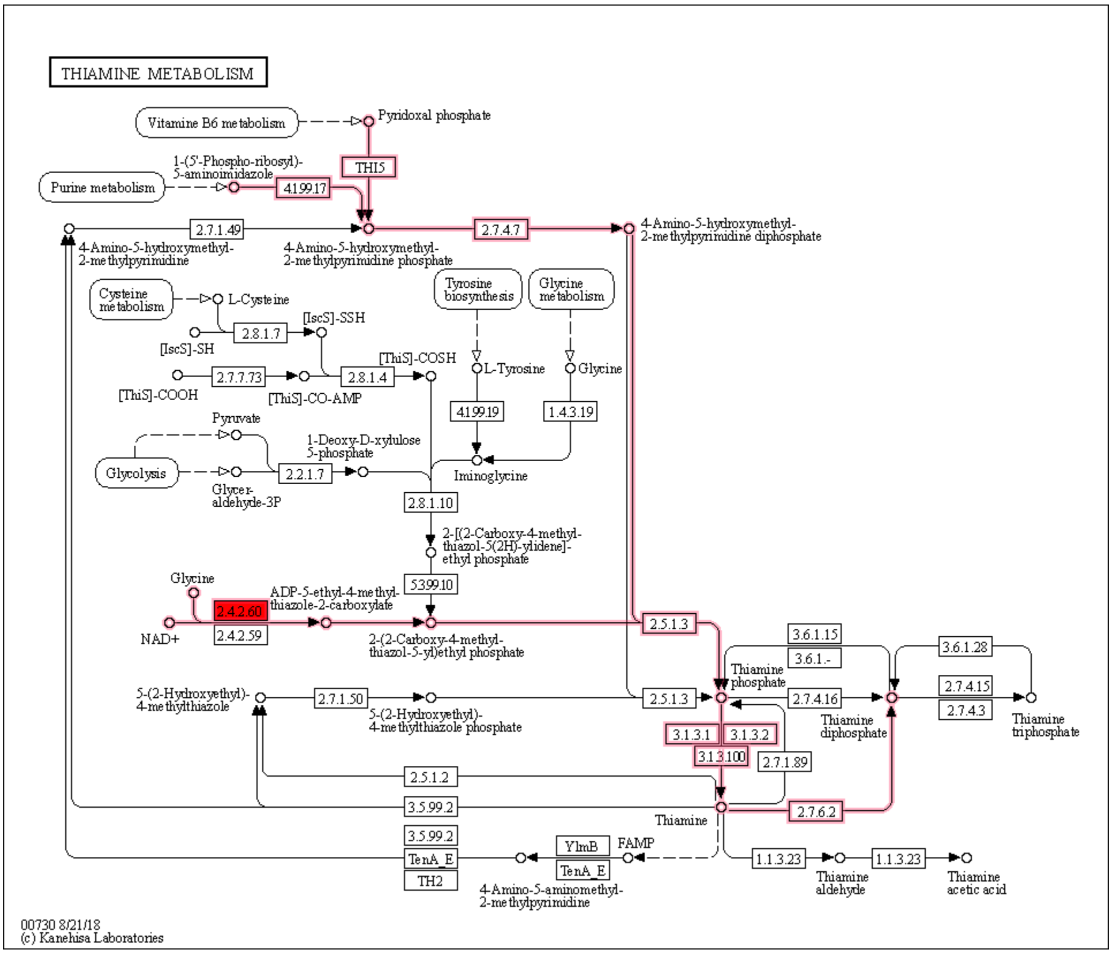

Supplementary Figure 4. Diagram of the thiamine metabolism pathway (KEGG map00730). K03146 was enriched after the IF intervention and labeled in red. The reactions relevant to thiamine synthesis which will be affected by K03146 are also highlighted.

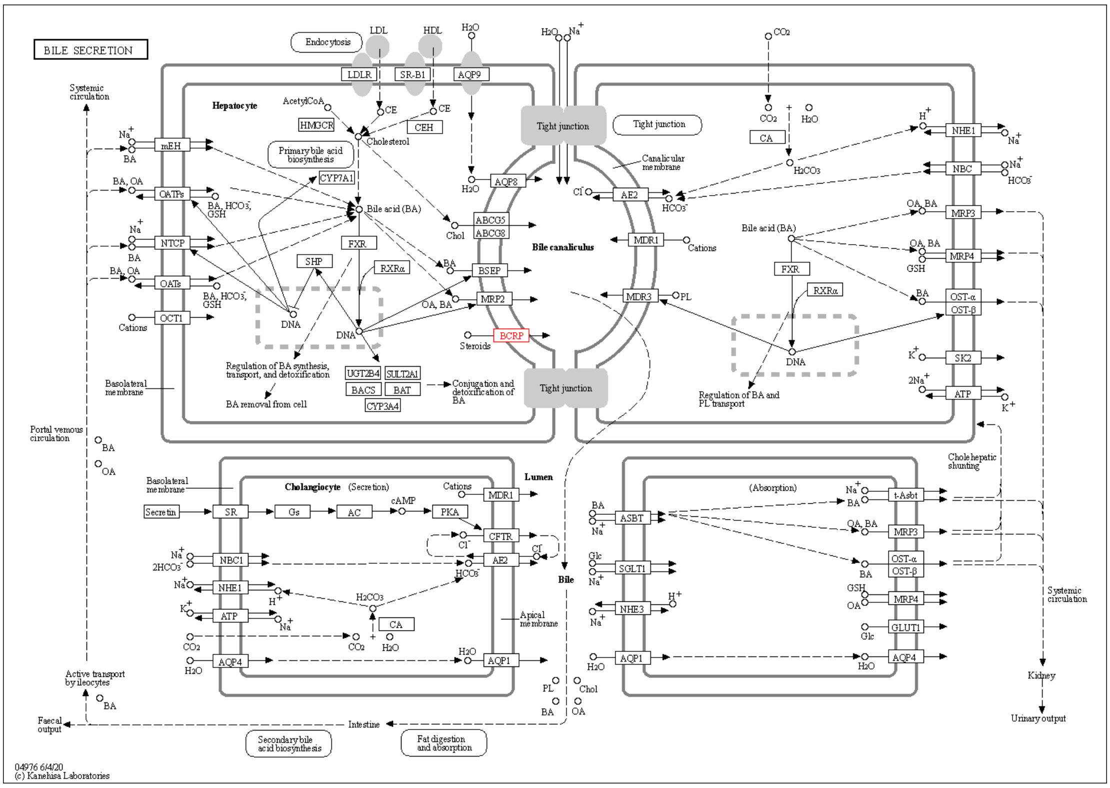

Supplementary Figure 5. Diagram of the bile secretion (KEGG map04976). K05681 was enriched after the IF intervention and labeled in red.

Supplementary Table 1. General information and clinical indices of the 72 participants.

SubjectID	Time point	Sex	SBP	DBP	AI	GLU	γ-GT	ALT	AST	LDL-C	TG	HDL-C	TC	UA	CREA	Weight	BFR	BMI	Waist
**S001**	Before	M	119	75	3.2	4.2	41	74.5	29.3	2.35	1.69	0.94	3.95	448	72	97.1	29.6	32.1	105.2
**S001**	After	M	105	64	1.11	3.9	21	45	22	1.69	0.85	0.92	2.79	500	91	89.8	28.1	29.6	98.7
**S003**	Before	M	146	91	4.61	4.17	34	38.7	24.7	3.48	4.01	1.14	6.4	479	82	103.2	33.2	35.7	110
**S003**	After	M	136	89	1.31	3.8	23	50	29	3.4	1.92	1.16	5.63	445	87	95.6	31.9	33.1	104
**S004**	Before	M	139	90	1.94	4.54	22	19.4	16.6	1.89	0.51	1.08	3.17	359	72	103.5	30.1	33.4	108
**S004**	After	M	114	73	1.34	3.9	18	29	17	1.61	1.81	1.02	3.17	427	86	98.7	29.6	31.9	101.6
**S005**	Before	M	127	87	3.55	4.38	28	71.9	34.6	3.39	2.01	1.19	5.41	577	72	88.2	28.9	30.5	107
**S005**	After	M	124	68	1.39	3.9	16	48	34	2.41	2.19	0.93	4.27	573	79	83.9	28	29	99.4
**S006**	Before	M	119	75	3.56	4.53	118	44.4	27.2	3.51	1.65	1.16	5.29	443	58	79.3	28	29.5	101
**S006**	After	M	103	61	1.13	4.6	90	52	24	2.93	1.06	1.19	4.5	436	65	77.2	27.1	28.7	91.4
**S007**	Before	M	116	70	3.33	4.31	24	19.4	13.7	2.93	1.06	0.99	4.29	392	78	91.7	29.9	31.7	98.2
**S007**	After	M	105	64	1.28	4.1	17	22	15	2.4	1.53	0.95	4.02	380	76	87.7	29.6	30.3	93.8
**S009**	Before	M	134	78	3.47	5.55	46	39.2	18.9	3.2	1.3	1.02	4.56	445	73	91	29.1	30	107.8
**S009**	After	M	114	75	1.13	3.8	27	48	29	2.2	0.71	0.93	3.41	593	82	83.8	26.8	27.7	98.5
**S010**	Before	M	130	72	4.83	5.46	38	24.1	16.2	3.88	3.4	0.98	5.71	431	84	104	32.1	34.3	108
**S010**	After	M	119	86	1.19	5	23	17	13	3.62	1.81	0.91	5.2	459	93	98.1	31.1	32.4	107.7
**S011**	Before	F	106	81	2.76	4.29	22	16.4	16.3	3.39	0.74	1.39	5.23	250	42	71.5	40.5	31.4	97
**S011**	After	F	108	77	1.21	4.2	17	16	15	2.4	1.18	1.05	3.96	239	48	70	40.2	30.7	96
**S012**	Before	F	145	99	2.77	4.35	17	8.9	12.4	3.14	1.56	1.33	5.02	415	69	72.7	34.5	27.4	95.5
**S012**	After	F	133	80	1.09	3.9	12	10	13	2.7	0.85	1.32	4.25	312	70	71.8	34.1	27	92
**S013**	Before	M	137	84	4	6.36	52	36	24.3	3.6	2.26	1.16	5.8	539	64	73.5	26.2	26.7	99.5
**S013**	After	M	118	72	1.13	5.3	29	40	34	3.32	0.85	1.38	5.12	509	74	67.5	23.6	24.5	92.7
**S014**	Before	M	146	103	2.84	4.83	68	86.7	38.2	4.39	2.46	1.77	6.8	534	74	81	29.2	29.4	100.6
**S014**	After	M	138	89	1.17	4.3	28	100	47	3.11	0.77	1.49	5.14	632	88	73.8	26.1	26.8	93.4
**S015**	Before	M	155	87	3.44	5.09	20	50.8	25.9	2.67	1.58	1	4.44	455	82	90.8	27.1	28.7	100.3
**S015**	After	M	128	76	1.27	3.8	15	30	19	2.5	1.37	0.88	4.06	546	79	85.5	24.4	27	94.5
**S016**	Before	M	173	109	5.52	6.89	87	68	50.8	3.39	5.49	0.92	6	444	68	85.6	28.1	28.3	95.4
**S016**	After	M	154	95	1.31	4.9	46	43	32	2.41	1.83	0.92	4.08	401	88	79.7	25.4	26.3	91.1
**S017**	Before	M	127	84	2.15	4.34	73	47.1	27.3	1.64	1.72	1.37	4.32	446	74	82	26.9	28	100
**S017**	After	M	123	83	1.85	4.1	23	13	15	1.07	1.19	1.33	3.31	488	88	75.4	25.5	25.8	93
**S019**	Before	F	118	75	2.47	4.96	26	30.4	16.9	3.02	0.97	1.38	4.79	279	56	66.2	36.5	27.2	99
**S019**	After	F	119	74	1.12	4.7	15	14	14	2.58	0.64	1.21	4.11	340	65	62.9	34.8	25.8	92.9
**S021**	Before	M	119	67	3.83	4.82	57	39.6	37.3	3.83	3.04	1.22	5.89	615	57	99.3	29.5	31	103.7
**S021**	After	M	119	73	1.17	4.4	33	39	37	3.55	1.33	1.27	5.41	547	59	96.4	28.4	30.1	101
**S022**	Before	M	122	87	2.94	4.71	49	89.1	50.5	3.42	2.15	1.35	5.32	361	61	71.7	28.7	28	99.8
**S022**	After	M	115	79	1.12	4.8	33	63	39	3.37	1.59	1.35	5.11	308	64	69.7	27.6	27.2	93
**S023**	Before	F	100	72	3.1	4.7	16	28	23.4	3.13	1.17	1.14	4.67	351	46	72.8	37.8	27.4	90.5
**S023**	After	F	94	61	1.16	4.4	11	19	17	2.48	0.96	0.95	3.83	320	50	69.2	36.5	26	86.5
**S025**	Before	F	108	73	2.61	5.1	29	33.6	20.1	3.2	0.58	1.41	5.09	395	61	73.3	38.2	27.9	96
**S025**	After	F	107	72	1.16	5.9	15	21	16	2.64	0.65	1.51	4.56	289	63	70.5	36.4	26.9	90.1
**S026**	Before	F	136	88	2.86	4.08	23	15.2	16.5	1.31	3.75	1.02	3.94	299	61	61.9	35.4	27.9	91.5
**S026**	After	F	137	85	1.4	4	14	13	15	2.14	2	1.25	4.24	292	71	58.4	34.2	26.3	87
**S027**	Before	M	114	63	3.05	4.59	31	22.4	19.5	3.11	1.39	1.18	4.78	355	66	81.6	23.9	26.9	93
**S027**	After	M	106	67	1.18	4.3	17	16	14	2.23	0.96	0.95	3.59	362	75	75.2	22	24.8	82.8
**S031**	Before	M	112	67	3.63	4.52	40	105.9	49.9	2.74	2.95	1.14	5.28	507	76	80.8	24.9	26.7	95.8

**S031**	After	M	107	66	1.27	4.4	15	28	30	2.02	1.2	1.27	3.83	456	89	73.5	23.4	24.3	86.5
**S033**	Before	F	121	75	1.99	5.24	26	16	16.1	2.88	0.96	1.59	4.75	184	46	71.1	36.3	27.8	94
**S033**	After	F	130	80	1.19	4	19	16	18	2.27	0.59	1.3	4.01	249	59	66.8	34.7	26.1	88.5
**S035**	Before	F	97	57	2.74	4.91	16	11.3	13.2	4.16	0.33	1.6	5.99	259	50	79.8	39.3	29.7	89
**S035**	After	F	98	69	1.16	3.6	11	9	15	3.62	0.66	1.18	5.39	237	61	76.4	38.2	28.4	88.9
**S037**	Before	M	119	78	2.74	6.06	32	21.9	17.8	3.55	1.24	1.44	5.38	387	83	71.2	25.7	26.1	89.2
**S037**	After	M	125	79	1.19	4.3	20	19	19	3.02	1.33	1.29	4.89	348	89	69.2	25.6	25.4	84.8
**S038**	Before	F	122	75	2.97	4.79	37	60	43.1	2.55	2.28	1.16	4.61	618	59	73.2	36.2	26.6	95
**S038**	After	F	109	73	1.25	4.6	16	36	32	2.05	0.77	1.18	3.75	546	69	68.3	33.8	24.8	88.9
**S039**	Before	F	123	81	2.52	4.43	17	19.7	15.5	3	1.04	1.39	4.89	345	65	67.7	36.9	27.4	95
**S039**	After	F	125	95	1.2	3.5	12	16	16	2.38	0.95	1.25	4.1	415	80	61.5	33.2	24.9	86.5
**S040**	Before	M	136	95	3.13	6.33	38	60.5	38.5	2.99	2.03	1.14	4.71	442	72	86.4	26.2	26.1	103
**S040**	After	M	125	90	1.18	4.5	23	54	35	2.64	1.22	1.01	4.13	448	88	82.8	24.1	25	94.5
**S042**	Before	F	140	80	2.77	8.02	18	16.8	17	3.61	1.09	1.46	5.5	348	46	58.5	33.2	25.6	89.5
**S042**	After	F	132	84	1.11	6.1	11	11	18	3.82	0.68	1.43	5.67	325	53	55	29.6	24.1	88.8
**S044**	Before	F	114	65	1.84	4.67	13	9.4	12.1	2.56	0.5	1.52	4.31	300	51	68.6	34.4	25.2	88.5
**S044**	After	F	118	75	1.19	3.8	10	16	20	2.42	0.59	1.35	4.22	360	54	63	32.7	23.1	83.8
**S046**	Before	F	121	81	1.78	4.64	15	15.2	21.5	3.15	1.22	2.05	5.69	218	62	58.9	34.3	24.2	92.5
**S046**	After	F	128	78	1.09	4.5	13	19	19	3.07	0.58	1.88	5.22	237	61	56.4	32.3	23.2	88.2
**S047**	Before	M	151	113	4.05	4.16	28	14.7	17.5	4.54	1.08	1.26	6.36	618	87	69.4	22.4	24	88.5
**S047**	After	M	123	87	1.19	3.5	19	21	22	2.4	0.82	1.37	4.22	711	90	64.7	21.5	22.4	79.5
**S048**	Before	F	116	74	2.56	5.22	21	19.5	15.6	2.53	1.57	1.21	4.31	299	57	69.3	36.9	26.4	96
**S048**	After	F	111	76	1.2	4.3	17	16	15	2.22	1.2	1.07	3.73	263	65	66.4	34.7	25.3	94.7
**S050**	Before	M	93	56	3.19	5.32	34	16.5	15	2.71	2.89	1.13	4.74	422	76	72.4	23.4	26	87
**S050**	After	M	111	66	1.22	4.5	27	15	18	2.39	1.72	1.08	4	343	75	72	23.9	25.8	82.5
**S052**	Before	F	111	69	2.5	4.64	21	12.9	18.2	2.07	1.26	1.04	3.64	227	49	65.2	33	24.2	90.5
**S052**	After	F	100	72	1.17	4.1	14	9	14	2.11	0.54	1.09	3.55	300	56	60.5	30.6	22.5	85.2
**S054**	Before	M	121	70	3.26	4.69	35	67.8	24.9	4.03	0.57	1.34	5.71	389	76	74.2	21.3	24.8	93
**S054**	After	M	97	62	1.16	3.6	19	30	21	2.75	0.67	1.2	4.39	408	87	68.1	19.6	22.8	86.8
**S058**	Before	F	116	71	1.45	4.51	26	33.1	24.9	1.71	0.99	1.54	3.77	238	40	63.3	33.2	24.7	89
**S058**	After	F	108	61	1.25	4.3	19	25	16	1.62	0.71	1.17	3.19	232	42	62.5	32.8	24.4	85.8
**S059**	Before	F	105	67	2.25	3.87	10	10.3	16.4	2.81	1.2	1.55	5.04	273	56	58.1	33.5	23.6	88
**S059**	After	F	102	65	1.37	4	7	11	16	2.22	1.37	1.39	4.43	243	56	56.7	33	23	85.9
**S060**	Before	F	123	77	2.46	4.46	15	22.8	16.9	3.61	0.99	1.64	5.67	464	62	76.7	37.6	26.9	92.5
**S060**	After	F	113	76	1.15	3.9	10	17	15	2.53	0.74	1.31	4.21	358	69	71.7	34.9	25.1	90.6
**S067**	Before	M	119	67	2.23	4.1	26	21.6	15.4	2.09	0.58	1.06	3.42	374	70	84.5	26.7	28.2	91.2
**S067**	After	M	119	64	1.18	3.8	22	23	16	2.05	0.47	1.01	3.43	364	66	80.6	25	26.9	88
**S072**	Before	M	97	69	2.36	4.03	26	15.5	16.6	2.85	1.42	1.43	4.81	370	76	60.8	20.5	22.6	79.4
**S072**	After	M	101	77	1.24	4.4	17	10	13	2.62	1.39	1.41	4.66	337	81	59.2	20.8	22	75.4
**S073**	Before	F	128	78	2.05	4.47	16	15.4	16.7	2.16	1.7	1.34	4.09	342	47	56.1	32.9	23.1	86.5
**S073**	After	F	114	71	1.21	4.8	13	10	14	2.38	1.09	1.43	4.32	352	60	52.6	28.7	21.6	82.1
**S074**	Before	F	119	79	2.19	4.58	15	16.6	18.6	3.24	1.21	1.62	5.16	304	56	62	33	23.6	91
**S074**	After	F	109	65	1.14	4.1	9	12	18	2.21	0.6	1.34	3.86	303	58	59.8	29.1	22.8	84.6
**S077**	Before	M	129	75	2.3	4.32	15	17.3	18.6	2.43	1.17	1.23	4.06	448	72	76.8	20.8	23.2	87
**S077**	After	M	125	81	1.18	4	13	12	15	2.41	0.84	1.12	3.97	402	80	73.9	21.1	22.3	82.1
**S078**	Before	M	128	82	3.33	4.35	42	46.8	26.2	2.48	2.37	1.05	4.55	561	81	72.9	20	23	85.6
**S078**	After	M	106	79	1.21	4.1	18	20	15	1.97	0.81	1.03	3.41	508	87	68	18.1	21.5	79.6

**S079**	Before	F	93	57	2.75	3.64	10	11.2	17	3.7	1.11	1.53	5.74	290	51	56.3	33.5	22.8	79
**S079**	After	F	92	54	1.13	4.2	8	8	16	3.39	0.64	1.31	5.13	267	54	54.6	29.2	22.1	78.2
**S080**	Before	F	113	73	2.13	5.32	13	13.8	16.4	2.53	0.97	1.38	4.32	390	69	56.8	33.7	23.6	86
**S080**	After	F	99	72	1.15	5.2	11	9	15	2.73	0.72	1.3	4.45	376	77	53.9	30.7	22.4	80
**S081**	Before	F	112	71	2.02	4.72	12	13.6	14.5	2.86	0.81	1.67	5.04	307	48	55.5	27.7	22.8	88.5
**S081**	After	F	100	57	1.17	4.5	15	18	18	1.86	0.47	1.56	3.73	252	57	53.1	27.2	21.8	80.4
**S083**	Before	F	108	69	2.35	4.34	24	25.2	17.3	3.18	0.76	1.53	5.12	286	46	64.5	33.9	23.1	88.5
**S083**	After	F	104	72	1.15	4.1	11	17	21	2.54	0.62	1.38	4.31	291	61	60.5	30.2	21.7	85.9
**S084**	Before	M	133	80	2.29	4.73	42	31.3	20.2	3.07	0.7	1.54	5.06	397	72	65.5	22.3	22.9	88
**S084**	After	M	135	81	1.15	4.5	20	20	19	2.46	0.72	1.42	4.24	337	75	61.4	19.6	21.5	75.8
**S085**	Before	F	88	64	3.67	4.76	12	13.6	12.7	2.87	1.34	0.97	4.53	217	53	62.6	34.1	23.6	85
**S085**	After	F	98	69	1.28	4.4	11	10	14	2.4	1.89	0.97	4.05	209	60	60.2	28.7	22.6	80.7
**S086**	Before	F	136	82	2.02	4.42	22	19.1	17	1.97	0.67	1.22	3.68	361	57	66.7	34.2	23.3	84
**S086**	After	F	114	72	1.22	3.6	17	16	12	1.87	0.57	1.24	3.52	373	64	63.2	30.5	22.1	80.3
**S087**	Before	M	115	65	2.1	3.92	27	15.8	15.1	3.17	0.87	1.66	5.14	394	93	67.5	21.8	24.2	82.8
**S087**	After	M	119	67	1.07	4	20	16	16	3	0.61	1.35	4.56	417	84	65	21.2	23.3	81.7
**S088**	Before	F	113	65	1.4	4.51	15	17.3	31.6	1.45	0.38	1.31	3.15	268	54	57.4	32.9	23	84
**S088**	After	F	97	65	1.22	3.9	17	24	39	1.53	0.52	1.18	3.04	295	66	53.9	27.3	21.6	79.7
**S089**	Before	F	119	67	3.11	4.88	20	47.1	30	3.15	2.53	1.41	5.79	357	49	61.9	28	22.2	88.5
**S089**	After	F	112	68	1.47	5.2	15	34	29	2.15	1.72	1.32	4.47	288	48	60.1	27.9	21.8	84.8
**S091**	Before	F	91	62	2.03	4.47	15	13.3	13.5	2.93	0.51	1.6	4.85	219	42	55.5	28.1	22	81
**S091**	After	F	97	59	1.08	4.3	12	12	14	2.89	0.61	1.64	4.77	271	54	54	26.6	21.4	78.3
**S092**	Before	F	104	66	1.67	4.71	15	6.5	11.9	2.6	0.38	1.81	4.83	279	57	51.2	29	22.2	75
**S092**	After	F	107	68	1.15	4.3	11	7	10	2.57	0.6	1.55	4.51	354	62	48.5	28.1	21	67.9
**S093**	Before	M	114	60	2.65	4.13	25	52.9	28.5	2.7	0.85	1.28	4.67	422	66	65.8	21.1	23.9	83
**S093**	After	M	112	66	1.16	3.5	17	16	16	2.29	0.72	1.25	3.91	397	84	60.4	20.1	21.9	77.6
**S094**	Before	F	113	75	2.01	4.69	13	12.3	16.3	2.48	0.46	1.45	4.37	327	52	57	31.6	24	81
**S094**	After	F	134	88	1.28	4.6	10	18	19	1.78	0.53	1.13	3.4	327	62	52.9	29.5	22.3	74.1
**S095**	Before	M	122	63	2.17	5.06	16	14.3	20.7	3.28	1.28	1.72	5.46	368	85	74	19.9	22.6	76.5
**S095**	After	M	119	71	0.87	4.5	11	15	25	2.54	0.83	1.79	3.99	436	102	68.9	18.2	21	72
**S096**	Before	M	132	83	3.48	4.43	33	21.4	21.6	3.24	1.3	1.15	5.15	319	69	69.8	20.4	22	85
**S096**	After	M	126	80	1.14	4.3	21	11	15	3.1	1.23	1.15	4.69	321	80	66.6	18	21	81.2
**S098**	Before	M	116	64	2.5	3.69	20	24.3	20.1	2.34	2.76	1.25	4.37	465	72	68.7	18.5	22.2	80
**S098**	After	M	116	72	1.14	3.3	16	12	13	2.48	0.81	1.33	4.15	393	77	65.7	17.6	21.2	73.5
**S099**	Before	M	93	62	4.36	4.37	18	9.4	15	4.59	1.67	1.18	6.33	289	78	62.8	20.3	22.5	81.3
**S099**	After	M	112	79	1.12	4.4	15	8	14	4.76	1.36	1.09	6.42	274	84	60.4	19.1	21.6	76.3
**S100**	Before	F	110	73	1.63	4.13	12	15.4	15.8	2.84	0.3	2.05	5.39	261	56	53.9	29.7	22.4	79
**S100**	After	F	106	72	1.17	4.3	11	8	16	2.78	0.9	1.63	4.89	201	61	51.7	28.5	21.5	78.2
**S101**	Before	F	122	74	2.52	4.41	15	11.6	14.4	2.53	0.54	1.16	4.08	215	52	61.2	29.3	21.4	84
**S101**	After	F	112	66	1.14	4.5	13	8	10	2.58	0.61	1.09	4.04	229	57	57.7	27.4	20.2	75.7
**S102**	Before	F	121	71	2.71	7.11	15	18	15.9	3.09	0.99	1.4	5.2	294	46	52.3	29.7	22.3	84.5
**S102**	After	F	111	71	1.19	4.7	13	14	14	2.4	0.8	1.2	4.06	301	53	49.6	27.7	21.2	81.9
**S103**	Before	F	110	71	1.93	4.23	14	11.7	15.6	2.72	0.89	1.61	4.71	268	65	53.5	34.5	24.1	83
**S103**	After	F	107	66	1.25	4.1	10	17	19	2.31	0.66	1.52	4.41	264	67	51.2	32.8	23.1	79.4
**S105**	Before	F	100	67	1.74	3.93	18	12	16.9	2.93	0.71	1.88	5.16	264	53	54.8	34.4	23.7	85
**S105**	After	F	104	69	1.12	4.1	12	7	13	2.83	0.66	1.53	4.69	289	59	54	34.1	23.4	82.9
**S106**	Before	F	117	76	1.33	4.87	30	45.8	39.7	2.21	0.19	1.92	4.47	291	37	62.3	30.5	22.6	87.5

**S106**	After	F	100	61	1.14	4.9	16	12	18	2.44	0.53	1.67	4.44	266	44	60.7	29.1	22	81.4
**S108**	Before	F	120	80	3.18	4.66	21	40	20.4	3.87	1.24	1.37	5.73	275	57	52.4	29.5	22.4	86
**S108**	After	F	125	82	1.12	4.6	14	20	15	3.46	1.03	1.33	5.19	316	63	49.2	27.3	21	79.9
**S110**	Before	F	101	65	1.33	4.46	19	13.4	13.7	2.06	0.43	1.79	4.17	283	53	56.8	27.2	21.4	80.5
**S110**	After	F	109	70	1.16	4.9	15	15	15	2.76	0.58	1.87	5.08	313	57	54.9	27.1	20.7	72.9
**Paired Wilcoxon rank sum test**																			
	Adjusted *p*		5.60E-05	8.92E-02	7.20E-13	6.17E-08	1.22E-12	6.58E-05	6.76E-03	6.94E-09	3.30E-06	1.72E-06	6.37E-11	5.23E-01	2.69E-11	7.20E-13	1.50E-12	7.20E-13	7.20E-13
	Difference in average		-5.4306	-5.4306	-1.5283	-0.4385	-10.0000	-7.3139	-2.1333	-0.4004	-0.4339	-0.0983	-0.6297	-2.1528	7.0694	-3.6639	-1.7403	-1.3236	-5.0875

Supplementary Table 2. Change in relative abundances of MAGs in the 72 participants.

MAG[a]	Genus	Species	Before（Mean）	After（Mean）	Adjusted p
**S100C1769**	*Bacteroides*	*Bacteroides thetaiotaomicron*	7.30008E-05	0.000372234	0.01598741
**S63C7593**	*Agathobacter*	*Agathobacter rectalis*	0.002240079	0.001364009	0.002467678
**S57C738**	*Fusicatenibacter*	*Fusicatenibacter saccharivorans*	1.95296E-05	0.000161984	0.011765457
**S63C1476**	*Bacteroides*	*Bacteroides sp900066265*	6.87175E-05	0.000211669	0.001899874
**S61C36565**	*Clostridium_Q*	*Clostridium_Q sp003024715*	0.000235607	0.00025404	0.011970784
**S59C1769**	*Bacteroides*	*Bacteroides thetaiotaomicron*	2.62116E-05	6.78176E-05	0.01353257
**S61C2353**	*Bacteroides*	*Bacteroides intestinalis_A*	4.30053E-05	0.001511186	0.003984676
**S64C7241**	*Agathobacter*	*Agathobacter rectalis*	0.000455403	0.000154337	0.001514674
**S64C86**	*Bacteroides*	*Bacteroides caccae*	0.00654959	0.011907853	0.000220776
**S65C6386**	*Parabacteroides*	*Parabacteroides distasonis*	0.000240546	0.002414439	0.000332228
**S66C8800**	*Bacteroides*	*Bacteroides salyersiae*	2.03748E-05	0.000148508	0.032801047
**S112C939**	*Parabacteroides*	*Parabacteroides merdae*	0.000391355	0.00072219	0.001292565
**S113C2095**	*Bacteroides*	*Bacteroides cellulosilyticus*	0.000298633	0.000602805	0.003249089
**S113C22280**	*Agathobacter*	*Agathobacter rectalis*	0.000807915	0.001438825	0.004211167
**S68C2198**	*Bacteroides*	*Bacteroides cutis*	3.96124E-05	5.0643E-05	0.028824494
**S68C9412**	*Agathobacter*	*Agathobacter rectalis*	0.000873301	0.000727079	0.00083099
**S69C13350**	*Agathobacter*	*Agathobacter rectalis*	0.00029814	0.000132135	0.003101822
**S71C14073**	*Clostridium_Q*	*Clostridium_Q sp003024715*	0.000278425	0.000205905	0.002059014
**S71C724**	*Bacteroides*	*Bacteroides uniformis*	0.00233121	0.004463026	0.002467678
**S73C1476**	*Bacteroides*	*Bacteroides sp900066265*	0.000159716	0.000220412	0.023446809
**S73C1769**	*Bacteroides*	*Bacteroides thetaiotaomicron*	0.000374606	0.00036184	0.014780472
**S73C6386**	*Parabacteroides*	*Parabacteroides distasonis*	0.002220366	0.00733576	0.000224558
**S114C1769**	*Bacteroides*	*Bacteroides thetaiotaomicron*	0.000189222	0.000183712	0.017564702
**S75C835**	*Parabacteroides*	*Parabacteroides merdae*	0.000891896	0.001272442	0.002166136
**S76C6145**	*Parabacteroides*	*Parabacteroides distasonis*	0.000817594	0.001612255	0.000543004
**S77C368**	*Clostridium_Q*	*Clostridium_Q sp000435655*	6.69022E-05	1.57933E-05	0.027193958
**S79C2095**	*Bacteroides*	*Bacteroides cellulosilyticus*	0.000157734	0.000233919	0.024506996
**S80C835**	*Parabacteroides*	*Parabacteroides merdae*	0.000135423	0.000220893	0.027997835
**S116C6484**	*Agathobacter*	*Agathobacter rectalis*	0.005619072	0.001890015	0.024506996
**S82C209**	*Coprococcus*	*Coprococcus sp900066115*	0.000133634	0.000102187	0.01598741
**S83C1769**	*Bacteroides*	*Bacteroides thetaiotaomicron*	0.000172778	0.000188524	0.026402514
**S84C2095**	*Bacteroides*	*Bacteroides cellulosilyticus*	7.77331E-05	0.000396154	0.003993284
**S85C1769**	*Bacteroides*	*Bacteroides thetaiotaomicron*	0.000135706	0.00016078	9.39705E-05
**S85C724**	*Bacteroides*	*Bacteroides uniformis*	0.000846852	0.001689871	0.023873305
**S86C368**	*Clostridium_Q*	*Clostridium_Q sp000435655*	0.000494381	0.000187312	0.015569281
**S86C939**	*Parabacteroides*	*Parabacteroides merdae*	0.001043245	0.001333251	0.009138974
**S88C738**	*Fusicatenibacter*	*Fusicatenibacter saccharivorans*	1.50876E-05	4.19088E-05	0.005105991
**S89C11878**	*Agathobacter*	*Agathobacter rectalis*	0.000905648	0.000644703	0.00027162
**S89C835**	*Parabacteroides*	*Parabacteroides merdae*	0.000108849	0.000155049	0.032801047
**S90C2095**	*Bacteroides*	*Bacteroides cellulosilyticus*	6.93244E-05	0.000164519	0.030631855
**S91C1476**	*Bacteroides*	*Bacteroides sp900066265*	8.41216E-05	0.000179355	0.006147856
**S96C371**	*Bacteroides*	*Bacteroides intestinalis*	0.00012641	0.000799134	0.045592178
**S98C6141**	*Bacteroides*	*Bacteroides ovatus*	0.010495857	0.013103654	0.001955443
**S98C7783**	*Agathobacter*	*Agathobacter rectalis*	0.000686913	0.000110806	0.000423899
**S9C368**	*Clostridium_Q*	*Clostridium_Q sp000435655*	6.04987E-05	3.52581E-05	0.023873305
**S119C1769**	*Bacteroides*	*Bacteroides thetaiotaomicron*	8.95153E-05	0.000272064	0.001292565
**S11C428**	*Enterocloster*	*Enterocloster sp900541315*	0.000119596	5.15932E-05	0.027401063
**S11C6386**	*Parabacteroides*	*Parabacteroides distasonis*	0.000349389	0.000772887	0.00083099
**S11C7921**	*Clostridium_Q*	*Clostridium_Q sp003024715*	0.000265232	0.000181577	0.001000884
**S11C835**	*Parabacteroides*	*Parabacteroides merdae*	0.000244555	0.000343175	0.02736414
**S121C835**	*Parabacteroides*	*Parabacteroides merdae*	0.000256197	0.000636408	0.01598741
**S122C60**	*Bacteroides*	*Bacteroides salyersiae*	0.0011792	0.001407624	0.012505363
**S122C6386**	*Parabacteroides*	*Parabacteroides distasonis*	0.002714836	0.002954889	0.001514674
**S101C209**	*Coprococcus*	*Coprococcus sp900066115*	0.00039677	3.57521E-05	0.044348491
**S101C6157**	*Parabacteroides*	*Parabacteroides distasonis*	0.002200752	0.004235066	0.004605444
**S123C738**	*Fusicatenibacter*	*Fusicatenibacter saccharivorans*	9.34474E-05	0.000305849	0.034481088
**S124C6145**	*Parabacteroides*	*Parabacteroides distasonis*	0.000126439	0.000546555	9.39705E-05
**S125C1476**	*Bacteroides*	*Bacteroides sp900066265*	0.000340917	0.001146361	0.004848458
**S126C6145**	*Parabacteroides*	*Parabacteroides distasonis*	0.000241494	0.000312942	8.02525E-05
**S127C6386**	*Parabacteroides*	*Parabacteroides distasonis*	0.00053232	0.002331551	0.000306118
**S127C724**	*Bacteroides*	*Bacteroides uniformis*	0.000851665	0.001169703	0.021397394
**S129C9914**	*Agathobacter*	*Agathobacter rectalis*	0.000734589	0.000443707	0.000520153

**S130C6367**	*Bacteroides*	*Bacteroides ovatus*	0.007759963	0.010241539	0.00083099
**S130C724**	*Bacteroides*	*Bacteroides uniformis*	0.000674502	0.003276173	0.024506996
**S134C2353**	*Bacteroides*	*Bacteroides intestinalis_A*	0.000416015	0.001581104	0.001865064
**S135C9805**	*Agathobacter*	*Agathobacter rectalis*	0.000728805	0.000284957	0.00083099
**S13C6145**	*Parabacteroides*	*Parabacteroides distasonis*	0.001045013	0.002072799	0.000118135
**S140C1769**	*Bacteroides*	*Bacteroides thetaiotaomicron*	0.000192642	0.00074876	0.005199186
**S140C371**	*Bacteroides*	*Bacteroides intestinalis*	0.000332237	0.000377949	0.013568988
**S142C1769**	*Bacteroides*	*Bacteroides thetaiotaomicron*	0.00028109	0.000352233	0.003173116
**S143C2547**	*Parabacteroides*	*Parabacteroides goldsteinii*	3.37722E-05	0.000104252	0.047609565
**S145C2192**	*Bacteroides*	*Bacteroides uniformis*	0.000334108	0.000564668	0.02059654
**S145C835**	*Parabacteroides*	*Parabacteroides merdae*	0.000382558	0.000490444	0.011253409
**S146C13190**	*Agathobacter*	*Agathobacter rectalis*	0.0009278	0.000240845	0.000860599
**S147C8348**	*Clostridium_Q*	*Clostridium_Q sp003024715*	0.000562179	0.000338112	0.010509492
**S149C6289**	*Clostridium_Q*	*Clostridium_Q sp003024715*	0.000527061	0.000395656	0.007426627
**S149C738**	*Fusicatenibacter*	*Fusicatenibacter saccharivorans*	3.4942E-05	0.000103427	0.031890773
**S14C15**	*Bacteroides*	*Bacteroides faecis*	0.000432375	0.000768701	0.012505363
**S14C428**	*Enterocloster*	*Enterocloster sp900541315*	0.000244502	0.000165891	0.006827846
**S104C6386**	*Parabacteroides*	*Parabacteroides distasonis*	0.000227512	0.000590935	0.000857693
**S153C835**	*Parabacteroides*	*Parabacteroides merdae*	0.000268873	0.000389075	0.031874666
**S154C70621**	*Clostridium_Q*	*Clostridium_Q sp003024715*	0.000173254	0.000142945	0.04697612
**S154C738**	*Fusicatenibacter*	*Fusicatenibacter saccharivorans*	2.50581E-05	0.000256399	0.038774982
**S155C9316**	*Agathobacter*	*Agathobacter rectalis*	0.000620643	0.000312367	0.000857693
**S156C312**	*Bacteroides*	*Bacteroides bouchesdurhonensis*	7.27831E-05	0.000209818	0.00091452
**S156C724**	*Bacteroides*	*Bacteroides uniformis*	0.000138511	0.000247473	0.032801047
**S157C6561**	*Clostridium_Q*	*Clostridium_Q sp003024715*	0.0005472	0.000441301	0.023594097
**S158C2095**	*Bacteroides*	*Bacteroides cellulosilyticus*	2.72679E-05	0.000520582	0.003828378
**S158C6158**	*Agathobacter*	*Agathobacter rectalis*	0.00176407	0.000200427	0.00083099
**S15C6386**	*Parabacteroides*	*Parabacteroides distasonis*	0.000210368	0.001203899	0.000118135
**S16C1769**	*Bacteroides*	*Bacteroides thetaiotaomicron*	0.000160456	0.000240865	0.015526238
**S16C6386**	*Parabacteroides*	*Parabacteroides distasonis*	0.000455895	0.000776004	0.001422841
**S17C18344**	*Clostridium_Q*	*Clostridium_Q sp003024715*	0.000370294	0.000314641	0.001865064
**S18C209**	*Coprococcus*	*Coprococcus sp000154245*	0.000184516	2.74758E-05	0.015830748
**S22C9985**	*Agathobacter*	*Agathobacter rectalis*	0.001240043	0.000614186	0.00083099
**S24C6145**	*Parabacteroides*	*Parabacteroides distasonis*	0.000880596	0.003738895	9.39705E-05
**S25C738**	*Fusicatenibacter*	*Fusicatenibacter saccharivorans*	0.000111949	0.000441073	0.048855429
**S28C8083**	*Clostridium_Q*	*Clostridium_Q sp003024715*	0.000428259	0.000277203	0.00494398
**S2C209**	*Coprococcus*	*Coprococcus sp900066115*	0.000257891	0.000140004	0.028805886
**S30C1339**	*Clostridium*	*Clostridium paraputrificum*	4.34402E-05	9.81546E-07	0.012700445
**S105C8255**	*Agathobacter*	*Agathobacter rectalis*	0.000558803	8.49612E-05	0.001595066
**S109C428**	*Enterocloster*	*Enterocloster sp900541315*	0.000162154	0.000118571	0.022370818
**S30C368**	*Clostridium_Q*	*Clostridium_Q sp000435655*	8.97851E-05	1.59082E-05	0.003173116
**S30C69**	*Clostridium*	*Clostridium sp900540255*	5.19815E-05	1.2017E-05	0.004605444
**S31C1769**	*Bacteroides*	*Bacteroides thetaiotaomicron*	6.77988E-05	0.000259573	0.000423899
**S33C13678**	*Agathobacter*	*Agathobacter rectalis*	0.001345568	0.000177245	0.001514674
**S33C1769**	*Bacteroides*	*Bacteroides thetaiotaomicron*	6.85096E-05	0.000286808	0.001029532
**S33C371**	*Bacteroides*	*Bacteroides intestinalis*	5.19568E-05	0.000279782	0.042179859
**S33C738**	*Fusicatenibacter*	*Fusicatenibacter saccharivorans*	4.6572E-05	0.000141261	0.008976897
**S34C209**	*Coprococcus*	*Coprococcus sp900066115*	0.000117361	6.14033E-06	0.024506996
**S34C2353**	*Bacteroides*	*Bacteroides intestinalis_A*	2.84487E-05	0.000219534	0.020562582
**S34C6145**	*Parabacteroides*	*Parabacteroides distasonis*	0.000347807	0.00138524	0.00027162
**S34C835**	*Parabacteroides*	*Parabacteroides merdae*	0.000224986	0.000433849	0.006827846
**S35C9678**	*Agathobacter*	*Agathobacter rectalis*	0.001300415	0.001533301	0.005466978
**S37C6578**	*Clostridium_Q*	*Clostridium_Q sp003024715*	0.000417835	0.000239768	0.004165835
**S39C428**	*Enterocloster*	*Enterocloster sp900541315*	0.000840649	0.000315495	0.028805886
**S3C428**	*Enterocloster*	*Enterocloster sp900541315*	0.000362621	0.000119693	0.022656173
**S109C9291**	*Clostridium_Q*	*Clostridium_Q sp003024715*	0.000281242	0.000212411	0.00297912
**S10C7613**	*Clostridium_Q*	*Clostridium_Q sp003024715*	0.000237152	0.000171296	0.002467678
**S40C1769**	*Bacteroides*	*Bacteroides thetaiotaomicron*	2.30399E-05	9.32745E-05	0.030631855
**S40C2353**	*Bacteroides*	*Bacteroides intestinalis_A*	0.000150006	0.001327031	0.001549156
**S41C69**	*Clostridium*	*Clostridium sp900540255*	3.53119E-05	1.46166E-05	0.011201979
**S42C1769**	*Bacteroides*	*Bacteroides thetaiotaomicron*	0.000535295	0.001907784	0.004605444
**S42C209**	*Coprococcus*	*Coprococcus sp900066115*	0.000538016	4.70344E-06	0.004211167
**S43C738**	*Fusicatenibacter*	*Fusicatenibacter saccharivorans*	1.41503E-05	5.87168E-05	0.01598741
**S44C50442**	*Agathobacter*	*Agathobacter rectalis*	0.000891869	0.000100217	0.001029532

**S44C69**	*Clostridium*	*Clostridium sp900540255*	0.000315602	9.35124E-06	0.002040661
**S45C1769**	*Bacteroides*	*Bacteroides thetaiotaomicron*	0.000398152	0.002203307	0.000166019
**S46C1769**	*Bacteroides*	*Bacteroides thetaiotaomicron*	3.45723E-05	6.53593E-05	0.008976897
**S46C724**	*Bacteroides*	*Bacteroides uniformis*	0.000135798	0.000346591	0.027997835
**S48C12631**	*Agathobacter*	*Agathobacter rectalis*	0.000354608	0.000142046	0.006117832
**S49C2095**	*Bacteroides*	*Bacteroides cellulosilyticus*	4.82265E-05	0.00015749	0.008743905
**S49C2192**	*Bacteroides*	*Bacteroides uniformis*	7.19921E-05	0.000362805	0.01984461
**S49C368**	*Clostridium_Q*	*Clostridium_Q sp000435655*	3.22759E-05	1.00957E-05	0.008976897
**S49C835**	*Parabacteroides*	*Parabacteroides merdae*	0.000169005	0.000265928	0.019853687
**S50C6145**	*Parabacteroides*	*Parabacteroides distasonis*	0.000435141	0.001897089	0.000118135
**S50C6484**	*Agathobacter*	*Agathobacter rectalis*	0.003896331	0.001921642	0.012334314
**S110C6247**	*Agathobacter*	*Agathobacter rectalis*	0.001873402	0.002099307	0.014571498
**S110C738**	*Fusicatenibacter*	*Fusicatenibacter saccharivorans*	7.74035E-05	0.000135513	0.024506996
**S111C428**	*Enterocloster*	*Enterocloster sp900541315*	0.000106249	3.48001E-05	0.027706354
**S111C6145**	*Parabacteroides*	*Parabacteroides distasonis*	0.000146226	0.000753269	8.02525E-05
**S50C738**	*Fusicatenibacter*	*Fusicatenibacter saccharivorans*	0.000183434	0.000374836	0.047960996
**S52C18268**	*Agathobacter*	*Agathobacter rectalis*	0.000632326	0.000156832	0.003173116
**S52C209**	*Coprococcus*	*Coprococcus sp900066115*	0.000187145	5.06298E-06	0.030631855
**S53C2095**	*Bacteroides*	*Bacteroides cellulosilyticus*	7.32796E-05	0.000939288	0.012497911
**S55C1769**	*Bacteroides*	*Bacteroides thetaiotaomicron*	8.79889E-05	0.000291099	0.002675355
**S57C6145**	*Parabacteroides*	*Parabacteroides distasonis*	0.000462064	0.000523968	0.000320384

[a]Only 147 MAGs with annotation at the species level are listed.

Supplementary Table 3. Correlation coefficient between abundance of MAGs and phenotype.

MAG	Genus	Species	DBP	AI	ALT	LDL-C	UA	Weight	BFR	Waist
**S100C1769**	*Bacteroides*	*Bacteroides thetaiotaomicron*	0	0	0	0	0	0.029628574	0	0
**S63C1476**	*Bacteroides*	*Bacteroides sp900066265*	0	-0.0752223	0	-0.09219765	0	0	0	0
**S61C36565**	*Clostridium_Q*	*Clostridium_Q sp003024715*	0	-0.00134592	0	-0.03275658	0	0	0	0
**S61C2353**	*Bacteroides*	*Bacteroides intestinalis_A*	0	-0.00313635	0	-0.0253936	0	0	0	0
**S61C22**	*Alistipes_A*	*Alistipes_A indistinctus*	0	0	0	-0.01910435	0	0	0	0
**S63C7593**	*Agathobacter*	*Agathobacter rectalis*	0	0	0	-0.03145269	0	0	0	0
**S64C7241**	*Agathobacter*	*Agathobacter rectalis*	0	0	0	-0.00246821	0	0	0	0
**S64C86**	*Bacteroides*	*Bacteroides caccae*	0	-0.00977378	0	0	0	0	0	0
**S65C6386**	*Parabacteroides*	*Parabacteroides distasonis*	0	-0.0569159	0	-0.06137491	0	0	0	0
**S66C8800**	*Bacteroides*	*Bacteroides salyersiae*	0	-0.00130977	0	-0.07272683	0	0	-0.090159	0
**S68C1778**	*Alistipes*	*Alistipes onderdonkii*	0	0	0	-0.00790241	0	0	0	0
**S112C939**	*Parabacteroides*	*Parabacteroides merdae*	0	-0.07217073	0	-0.08362431	0	0	0	0
**S113C22280**	*Agathobacter*	*Agathobacter rectalis*	0	-0.00153094	0	-0.0558914	0	0	0	0
**S68C2198**	*Bacteroides*	*Bacteroides cutis*	0	-0.13496001	0	-0.14330746	0	0	0	0
**S68C9412**	*Agathobacter*	*Agathobacter rectalis*	0	0	0	-0.0272511	0	0	0	0
**S69C13350**	*Agathobacter*	*Agathobacter rectalis*	0	0	0	-0.02948632	0	0	0	0
**S71C14073**	*Clostridium_Q*	*Clostridium_Q sp003024715*	0	-0.00926522	0	-0.04029467	0	0	0	0
**S71C724**	*Bacteroides*	*Bacteroides uniformis*	0	-0.01492262	0	-0.05186833	0	0	0	0
**S73C1476**	*Bacteroides*	*Bacteroides sp900066265*	0	-0.1297162	0	-0.09399501	0	0	0	0
**S73C1769**	*Bacteroides*	*Bacteroides thetaiotaomicron*	0	0	0	-0.04402337	0	0	0	0
**S73C6386**	*Parabacteroides*	*Parabacteroides distasonis*	0	-0.0315334	0	-0.03693092	0	0	0	0
**S114C1769**	*Bacteroides*	*Bacteroides thetaiotaomicron*	0	0	0	-0.00785012	0	0	0	0
**S75C835**	*Parabacteroides*	*Parabacteroides merdae*	0	0	0	-0.03988984	0	0	0	0
**S76C1778**	*Alistipes*	*Alistipes onderdonkii*	0	-0.07529628	0	-0.06322517	0	0	0	0
**S76C6145**	*Parabacteroides*	*Parabacteroides distasonis*	0	-0.15134838	0	-0.09408403	0	-0.27310219	0	0
**S77C130**	*Phocaeicola*	*Phocaeicola coprocola*	0	-0.00981577	0	-0.04723229	0	0	0	0
**S80C290**	*Odoribacter*	*Odoribacter splanchnicus*	0	-0.04512286	0	-0.02425355	0	0	0	0
**S80C835**	*Parabacteroides*	*Parabacteroides merdae*	0	0	0	0	0	0.123861048	0	0
**S81C740**	*Dorea*	*Dorea longicatena*	0	0	0	0	0	-0.79026557	0	0
**S116C6484**	*Agathobacter*	*Agathobacter rectalis*	0	0	0	-0.01266994	0	0	0	0
**S82C8627**	*Zag111*	*Zag111 sp002103105*	0	0	0.16494899	-0.02950975	0.143987478	0	0	0
**S83C1769**	*Bacteroides*	*Bacteroides thetaiotaomicron*	0	0	0	0	0	0.844389919	0	0.376745
**S85C1769**	*Bacteroides*	*Bacteroides thetaiotaomicron*	0	-0.0154052	0	-0.05050548	0	0	0	0
**S85C724**	*Bacteroides*	*Bacteroides uniformis*	0	-0.07128656	0	-0.04565196	0	0	0	0
**S86C368**	*Clostridium_Q*	*Clostridium_Q sp000435655*	0	-0.01484974	0	-0.03581484	0	0	0	0
**S86C939**	*Parabacteroides*	*Parabacteroides merdae*	0	-0.12512522	0	-0.0888205	0	0	0	0
**S89C835**	*Parabacteroides*	*Parabacteroides merdae*	0	0	0	0	0.186400136	0	0	0
**S117C724**	*Bacteroides*	*Bacteroides uniformis*	0	-0.12820783	0	-0.08725764	0	0	0	0

**S118C570**	*Turicibacter*	*Turicibacter sp001543345*	0	-0.02689058	0	-0.03280738	0	0	0	0
**S91C1476**	*Bacteroides*	*Bacteroides sp900066265*	0	-0.10327681	0	-0.08718838	0	0	0	0
**S98C7783**	*Agathobacter*	*Agathobacter rectalis*	0	0	0	-0.04509489	0	0	0	0
**S119C1769**	*Bacteroides*	*Bacteroides thetaiotaomicron*	0	-0.00111176	0	-0.04276217	0	0	0	0
**S11C6386**	*Parabacteroides*	*Parabacteroides distasonis*	0	-0.00081069	0	-0.04494605	0	0	0	0
**S11C7921**	*Clostridium_Q*	*Clostridium_Q sp003024715*	0	-0.00206878	0	-0.03368452	0	0	0	0
**S121C835**	*Parabacteroides*	*Parabacteroides merdae*	0	-4.7215E-05	0	-0.02023497	0	0	0	0
**S122C60**	*Bacteroides*	*Bacteroides salyersiae*	0	-0.1358457	0	-0.08031518	0	0	0	0
**S122C6386**	*Parabacteroides*	*Parabacteroides distasonis*	0	-0.07704318	0	-0.07240177	0	0	0	0
**S123C253**	*Alistipes*	*Alistipes shahii*	0	-0.03725751	0	-0.03354708	0	0	0	0
**S123C54**	*Anaerobutyricum*	*Anaerobutyricum hallii_A*	0	0	0	0	0	0.369425786	0	0
**S101C6157**	*Parabacteroides*	*Parabacteroides distasonis*	0	-0.11918935	0	-0.07422443	0	0	0	0
**S123C738**	*Fusicatenibacter*	*Fusicatenibacter saccharivorans*	0	-0.01249221	0	-0.06246666	0	0	0	0
**S124C6145**	*Parabacteroides*	*Parabacteroides distasonis*	0	0	0	-0.05219062	0	0	0	0
**S125C1476**	*Bacteroides*	*Bacteroides sp900066265*	0	-0.04584126	0	-0.08037685	0	0	0	0
**S126C6145**	*Parabacteroides*	*Parabacteroides distasonis*	0	-0.03762479	0	-0.07159355	0	0	0	0
**S127C6386**	*Parabacteroides*	*Parabacteroides distasonis*	0	-0.04891208	0	-0.06191352	0	0	0	0
**S127C724**	*Bacteroides*	*Bacteroides uniformis*	0	-0.09130372	0	-0.0708963	0	0	0	0
**S128C334**	*Blautia_A*	*Blautia_A sp000436615*	0	-0.00058872	0	0	0	0	0	0
**S129C9914**	*Agathobacter*	*Agathobacter rectalis*	0	0	0	-0.02484294	0	0	0	0
**S130C724**	*Bacteroides*	*Bacteroides uniformis*	0	-0.05522488	0	-0.04133707	0	0	0	0
**S133C442**	*Roseburia*	*Roseburia intestinalis*	0	0	0	0	0	-0.17034607	0	0
**S134C2353**	*Bacteroides*	*Bacteroides intestinalis_A*	0	-0.08540159	0	-0.08463531	0	0	0	0
**S135C9805**	*Agathobacter*	*Agathobacter rectalis*	0	0	0	-0.00640123	0	0	0	0
**S13C6145**	*Parabacteroides*	*Parabacteroides distasonis*	0	-0.08074366	0	-0.06740355	0	0	0	0
**S140C1769**	*Bacteroides*	*Bacteroides thetaiotaomicron*	0	-0.02253206	0	-0.06316493	0	0	0	0
**S140C33**	*Erysipelatoclostridium*	*Erysipelatoclostridium ramosum*	0	-0.04858637	0	-0.08475725	0	0	0	0
**S142C1769**	*Bacteroides*	*Bacteroides thetaiotaomicron*	0	-0.00189081	0	-0.05009186	0	0	0	0
**S143C2547**	*Parabacteroides*	*Parabacteroides goldsteinii*	0	0	0	-0.00757721	0	0	0	0
**S145C2192**	*Bacteroides*	*Bacteroides uniformis*	0	-0.10694619	0	-0.08847773	0	0	0	0
**S145C835**	*Parabacteroides*	*Parabacteroides merdae*	0	-0.0016839	0	-0.02785158	0	0	0	0
**S146C13190**	*Agathobacter*	*Agathobacter rectalis*	0	0	0	-0.01542589	0	0	0	0
**S147C146**	*CAG-180*	*CAG-180 sp000432435*	0.12144194	0	0	0	0.173277899	0.425152706	0	0.300995
**S147C8348**	*Clostridium_Q*	*Clostridium_Q sp003024715*	0	-4.7782E-05	0	-0.01796317	0	0	0	0
**S149C6289**	*Clostridium_Q*	*Clostridium_Q sp003024715*	0	-0.00045434	0	-0.02431822	0	0	0	0
**S149C738**	*Fusicatenibacter*	*Fusicatenibacter saccharivorans*	0	0	0	0	0	0.224848184	0	0.191455
**S14C15**	*Bacteroides*	*Bacteroides faecis*	0	-0.0676851	0	-0.08239933	0	0	0	0
**S14C1777**	*Allisonella*	*Allisonella histaminiformans*	0	0	0	0	0	0	-0.177697	0
**S14C428**	*Enterocloster*	*Enterocloster sp900541315*	0	0	0	-0.02607857	0	0	0	0
**S104C6386**	*Parabacteroides*	*Parabacteroides distasonis*	0	-0.07168522	0	-0.07994208	0	0	0	0

**S153C835**	*Parabacteroides*	*Parabacteroides merdae*	0	0	0	-0.01952635	0	0	0	0
**S154C70621**	*Clostridium_Q*	*Clostridium_Q sp003024715*	0	-0.00615216	0	-0.04107949	0	0	0	0
**S155C9316**	*Agathobacter*	*Agathobacter rectalis*	0	0	0	-0.02829675	0	0	0	0
**S156C18468**	*UMGS1688*	*UMGS1688 sp900545885*	0.106341852	0	0.348816957	-0.03652417	0.270709087	0	0	0
**S156C312**	*Bacteroides*	*Bacteroides bouchesdurhonensis*	0	-0.07939773	0	-0.09020448	0	0	0	0
**S156C724**	*Bacteroides*	*Bacteroides uniformis*	0	-0.12677525	0	-0.08257979	0	0	0	0
**S157C6561**	*Clostridium_Q*	*Clostridium_Q sp003024715*	0	-0.0003892	0	-0.02488251	0	0	0	0
**S15C6386**	*Parabacteroides*	*Parabacteroides distasonis*	0	-0.11455045	0	-0.07947737	0	0	0	0
**S16C1769**	*Bacteroides*	*Bacteroides thetaiotaomicron*	0	-0.02480626	0	-0.05870033	0	0	0	0
**S16C6386**	*Parabacteroides*	*Parabacteroides distasonis*	0	-0.02969125	0	-0.0603727	0	0	0	0
**S17C18344**	*Clostridium_Q*	*Clostridium_Q sp003024715*	0	-0.00165384	0	-0.03080303	0	0	0	0
**S22C9985**	*Agathobacter*	*Agathobacter rectalis*	0	-0.03364598	0	-0.06824198	0	0	0	0
**S23C835**	*Parabacteroides*	*Parabacteroides merdae*	0	0	0	-0.02148631	0	0	0	0
**S24C6145**	*Parabacteroides*	*Parabacteroides distasonis*	0	-0.08852444	0	-0.07353577	0	0	0	0
**S28C8083**	*Clostridium_Q*	*Clostridium_Q sp003024715*	0	-0.00276592	0	-0.0356961	0	0	0	0
**S105C8255**	*Agathobacter*	*Agathobacter rectalis*	0	-0.02509155	0	-0.066036	0	0	0	0
**S109C411**	*Phascolarctobacterium*	*Phascolarctobacterium faecium*	0	0	0	-0.009222	0	0	0	0
**S109C428**	*Enterocloster*	*Enterocloster sp900541315*	0	0	0	-0.02473831	0	0	0	0
**S30C1724**	*Negativibacillus*	*Negativibacillus massiliensis*	0	-0.07538111	0	-0.08470014	0	0	0	0
**S31C1769**	*Bacteroides*	*Bacteroides thetaiotaomicron*	0	0	0	-0.03110533	0	0	0	0
**S31C253**	*Alistipes*	*Alistipes shahii*	0	0	0	-0.00105833	0	0	0	0
**S31C334**	*Blautia_A*	*Blautia_A sp000436615*	0	-0.04216018	0	-0.06613735	0	0	0	0
**S33C13678**	*Agathobacter*	*Agathobacter rectalis*	0	0	0	-0.00494289	0	0	0	0
**S33C1769**	*Bacteroides*	*Bacteroides thetaiotaomicron*	0	0	0	-0.01752611	0	0	0	0
**S33C5652**	*Acutalibacter*	*Acutalibacter sp900543555*	0	-0.00342821	0	-0.05778348	0	0	0	0
**S34C6145**	*Parabacteroides*	*Parabacteroides distasonis*	0	-0.03058673	0	-0.07161442	0	0	0	0
**S34C835**	*Parabacteroides*	*Parabacteroides merdae*	0	-0.00022823	0	-0.01813402	0	0	0	0
**S35C9678**	*Agathobacter*	*Agathobacter rectalis*	0	-0.03866881	0	-0.07479761	0	0	0	0
**S36C290**	*Odoribacter*	*Odoribacter splanchnicus*	0	0	0	-0.00116631	0	0	0	0
**S37C6578**	*Clostridium_Q*	*Clostridium_Q sp003024715*	0	-0.00202727	0	-0.02802584	0	0	0	0
**S39C1476**	*Bacteroides*	*Bacteroides sp900066265*	0	-0.11396787	0	-0.09469007	0	0	0	0
**S39C428**	*Enterocloster*	*Enterocloster sp900541315*	0	0	0	-0.0009616	0	0	0	0
**S39C6770**	*Blautia_A*	*Blautia_A massiliensis*	0	-0.02020323	0	-0.04764337	0	0	0	0
**S109C9291**	*Clostridium_Q*	*Clostridium_Q sp003024715*	0	-0.00168692	0	-0.02969465	0	0	0	0
**S10C7613**	*Clostridium_Q*	*Clostridium_Q sp003024715*	0	-0.00558255	0	-0.04404522	0	0	0	0
**S40C2353**	*Bacteroides*	*Bacteroides intestinalis_A*	0	-0.00374136	0	-0.02845638	0	0	0	0
**S42C1769**	*Bacteroides*	*Bacteroides thetaiotaomicron*	0	-0.0268407	0	-0.05986936	0	0	0	0
**S42C209**	*Coprococcus*	*Coprococcus sp900066115*	0	0	0	0	0	0	-0.191124	0
**S44C50442**	*Agathobacter*	*Agathobacter rectalis*	0	0	0	-0.01384833	0	0	0	0
**S44C69**	*Clostridium*	*Clostridium sp900540255*	0	0	0	0	0	-0.03004017	0	0

**S45C1769**	*Bacteroides*	*Bacteroides thetaiotaomicron*	0	-0.0551167	0	-0.04969716	0	0	0	0
**S46C1769**	*Bacteroides*	*Bacteroides thetaiotaomicron*	0	0	0	0	0.083783003	0.594590551	0	0
**S46C724**	*Bacteroides*	*Bacteroides uniformis*	0	-0.07341444	0	-0.07740718	0	0	0	0
**S47C74**	*CAG-95*	*CAG-95 sp000438155*	0	0	0	0	0.117871086	0	0	0
**S48C12631**	*Agathobacter*	*Agathobacter rectalis*	0	-0.00163725	0	-0.05115143	0	0	0	0
**S49C2095**	*Bacteroides*	*Bacteroides cellulosilyticus*	0	0	0	0	0	-0.49330047	0	0
**S49C2192**	*Bacteroides*	*Bacteroides uniformis*	0	-0.06238972	0	-0.0806724	0	0	0	0
**S49C835**	*Parabacteroides*	*Parabacteroides merdae*	0	-9.4915E-05	0	-0.04029729	0	0	0	0
**S50C6145**	*Parabacteroides*	*Parabacteroides distasonis*	0	-0.10591883	0	-0.08133768	0	0	0	0
**S50C6484**	*Agathobacter*	*Agathobacter rectalis*	0	-0.03955931	0	-0.07625877	0	0	0	0
**S110C6247**	*Agathobacter*	*Agathobacter rectalis*	0	0	0	-0.02077578	0	0	0	0
**S111C6145**	*Parabacteroides*	*Parabacteroides distasonis*	0	-0.06333993	0	-0.08519958	0	0	0	0
**S50C738**	*Fusicatenibacter*	*Fusicatenibacter saccharivorans*	0	0	0	-0.00022977	0	0	0	0
**S52C18268**	*Agathobacter*	*Agathobacter rectalis*	0	0	0	-0.0107385	0	0	0.178745	0
**S52C286**	*Citrobacter*	*Citrobacter freundii*	0	0	0	0	0	-0.3895382	0	0
**S55C1769**	*Bacteroides*	*Bacteroides thetaiotaomicron*	0	-0.06315708	0	-0.07985688	0	0	0	0
**S57C334**	*Blautia_A*	*Blautia_A sp000436615*	0	-0.00226213	0	-0.02093247	0	0	0	0
**S57C6145**	*Parabacteroides*	*Parabacteroides distasonis*	0	-0.10123685	0	-0.08098121	0	0	0	0

Supplementary Table 4. Correlation coefficient between abundance of KOs and phenotype.

KO	AI	γ-GT	AST	LDL-C	TG	TC	BFR
**K00011**	0	-0.041502	0	-0.238971	-0.085047	0	0
**K00118**	0	0	0	-0.261229	-0.106945	0	-0.000428064
**K00012**	0	0	0	-0.162598	-0.051206	0	0
**K00891**	0	0	0	-0.161899	-0.047532	0	0
**K00015**	-0.006327873	0	0	-0.284664	-0.128793	0	-0.000401554
**K00042**	0	0	0	-0.25418	-0.098489	0	-0.000302906
**K00033**	0	0	0	-0.23252	-0.090006	0	-0.000130994
**K00036**	0	0	0	-0.236948	-0.094584	0	-0.000115141
**K00067**	0	0	0	-0.160462	-0.048586	0	0
**K01784**	0	0	0	-0.120292	-0.02491	0	0
**K06607**	-0.125441259	0	0	-0.404319	-0.165859	-0.437478024	-0.421075866
**K13013**	-0.007467665	0	0	-0.278305	-0.125969	0	-0.000293265
**K15237**	-0.056368631	0	0	-0.347597	-0.160751	-0.127570062	-0.000421669
**K00102**	-0.005886888	0	0	-0.276276	-0.123221	0	-0.000453398
**K00156**	-0.020906601	0	0	-0.293778	-0.132385	0	-0.000339757
**K00214**	-0.084828446	0	0	-0.346855	-0.177891	-0.150079246	-0.172496534
**K00281**	0	0	0	-0.220636	-0.083486	0	-9.09E-05
**K00294**	-0.03633498	0	0	-0.314565	-0.149112	-0.039994892	-0.000411796
**K00318**	-0.084784869	0	0	-0.352607	-0.18049	-0.164088894	-0.000648753
**K00329**	-0.019594977	0	0	-0.301421	-0.14035	-0.011246242	-0.000414613
**K02574**	0	0	0	-0.178379	-0.061025	0	0
**K00395**	0	0	0	-0.297241	-0.129096	-5.70E-05	-0.059621509
**K04719**	-0.083085963	0	0	-0.3605	-0.174461	-0.211878989	-0.428346643
**K00549**	0	0	0	-0.200564	-0.070544	0	-4.28E-05
**K02169**	0	0	0	-0.222641	-0.084902	0	-0.000108814
**K01271**	0	0	0	-0.170903	-0.055055	0	0
**K00936**	0	0	0	-0.034308	0	0	0
**K11527**	0	0	0	-0.163238	-0.052286	0	0
**K00603**	0	0	0	-0.240545	-0.095591	0	-0.000125214
**K00605**	0	0	0	-0.210558	-0.077783	0	-0.00010606
**K00680**	0	0	0	-0.086536	-0.005453	0	0
**K09458**	0	0	0	-0.164847	-0.049923	0	0
**K00648**	0	0	0	-0.156188	-0.045446	0	0
**K03335**	0	0	0	-0.223235	-0.090516	0	-9.94E-05
**K03827**	0	0	0	-0.200865	-0.074077	0	-8.41E-06
**K01951**	0	0	0	-0.154127	-0.045057	0	0
**K13006**	0	0	0	-0.262468	-0.112584	0	-0.000330617
**K13018**	-0.028571407	0	0	-0.300549	-0.139679	0	-0.21759578
**K13668**	-0.002789568	0	0	-0.281171	-0.12395	0	-0.276435499
**K00729**	-0.064131369	0	0	-0.341132	-0.169329	-0.13895205	-0.000621264
**K00996**	0	0	0	-0.219869	-0.083845	0	-0.000133684
**K00759**	0	0	0	-0.174225	-0.053659	0	0
**K00762**	0	0	0	-0.159589	-0.046763	0	0
**K14261**	-0.04060159	0	0	-0.319307	-0.146119	-0.067809048	-0.000509804
**K13010**	0	0	0	-0.222237	-0.090127	0	-0.000114544
**K13017**	-0.032933035	0	0	-0.301901	-0.142199	-0.004245924	-0.000538172
**K00844**	-0.017091281	0	0	-0.29249	-0.131354	0	-0.000345082
**K00903**	0	0	0	-0.164984	-0.052545	0	0
**K00908**	0	0	0	-0.222761	-0.092741	0	-0.000169924
**K00928**	0	0	0	-0.156143	-0.045451	0	0
**K02484**	0	0	0	-0.168449	-0.052044	0	0
**K07678**	0	0	0	-0.255614	-0.10804	0	-0.000140393
**K05962**	0	0	0	-0.235198	-0.100347	0	-0.000152474
**K07647**	-0.00895183	0	0	-0.283216	-0.127508	0	-0.000216802
**K07679**	0	0	0	-0.22146	-0.091333	0	-0.000134898
**K14978**	-0.086225021	0	0	-0.38936	-0.187599	-0.277239164	-0.02053593

**K07716**	-0.014115017	0	0	-0.289853	-0.131766	0	-0.000522014
**K10715**	-0.075771805	0	0	-0.338445	-0.163977	-0.120089767	-0.00062442
**K10125**	-0.065838039	0	0	-0.347782	-0.170703	-0.163089321	-0.367302959
**K11443**	-0.023940448	0	0	-0.341584	-0.127598	-0.082303571	0
**K01809**	0	0	0	-0.168898	-0.054333	0	0
**K01138**	0	0	0	-0.119988	-0.024502	0	0
**K05968**	0	0	0	-0.184753	-0.063522	0	0
**K01057**	0	0	0	-0.241111	-0.096291	0	-0.000143473
**K02439**	0	0	0	-0.284422	-0.117244	0	-0.011587822
**K01130**	0	0	0	-0.162468	-0.04878	0	0
**K01134**	0	0	0	-0.174419	-0.054395	0	0
**K01133**	0	0	0	-0.224854	-0.087008	0	-0.00019826
**K01135**	-0.051070294	0	0	-0.317724	-0.150766	-0.045043064	-0.000410053
**K05349**	0	0	0	-0.101667	-0.014341	0	0
**K01811**	0	0	0	-0.156109	-0.047201	0	0
**K01192**	0	0	0	-0.182872	-0.064375	0	0
**K01195**	0	0	0	-0.179577	-0.059691	0	0
**K07407**	0	0	0	-0.131729	-0.03307	0	0
**K01191**	0	0	0	-0.205088	-0.079704	0	0
**K01243**	0	0	0	-0.173833	-0.053787	0	0
**K01251**	0	0	0	-0.246671	-0.106475	0	-0.000210871
**K01303**	0	0	0	-0.191843	-0.066634	0	-2.86E-05
**K01337**	-0.057099424	0	0	-0.311909	-0.152953	-0.045886384	-0.000543792
**K01412**	0	0	0	-0.263178	-0.117217	0	-0.000247829
**K06194**	0	0	0	-0.196582	-0.067871	0	-6.21E-06
**K06894**	0	0	0	-0.188402	-0.064545	0	-4.60E-05
**K01476**	0	0	0	-0.268188	-0.1186	0	-0.000393453
**K01488**	0	0	0	-0.250504	-0.09786	0	-0.000222501
**K01958**	0	0	0	-0.256573	-0.108977	0	-0.000218175
**K01580**	0	0	0	-0.235176	-0.096131	0	-6.74E-05
**K01607**	0	0	0	-0.171865	-0.056809	0	0
**K15037**	-0.077538005	0	0	-0.366106	-0.172819	-0.195237158	-0.000563944
**K01623**	0	0	0	-0.22761	-0.08772	0	-0.000113483
**K01654**	0	0	0	-0.223105	-0.088557	0	-0.000166276
**K01666**	0	0	0	-0.235551	-0.093657	0	-0.000142556
**K01667**	0	0	0	-0.264016	-0.116462	0	-0.000238759
**K03780**	0	0	0	-0.273334	-0.111088	0	-0.000387978
**K01712**	0	0	0	-0.225798	-0.087422	0	-9.87E-05
**K01745**	0	0	0	-0.2071	-0.075228	0	-4.09E-05
**K01754**	0	0	0	-0.188603	-0.063043	0	-2.81E-05
**K05606**	0	0	0	-0.217023	-0.080421	0	-0.000142628
**K08234**	0	0	0	-0.238627	-0.092171	0	-0.000219555
**K01865**	-0.072952675	0	0	-0.354544	-0.179044	-0.177912433	-0.094744456
**K01791**	0	0	0	-0.168753	-0.055648	0	0
**K13685**	0	0	0	-0.207353	-0.077363	0	-0.000147714
**K01807**	0	0	0	-0.259065	-0.109431	0	-0.000236109
**K01816**	-0.009186137	0	0	-0.289838	-0.130959	0	-0.000359714
**K01843**	0	0	0	-0.231281	-0.091746	0	-9.87E-05
**K01847**	0	0	0	-0.181233	-0.059248	0	0
**K09181**	0	0	0	-0.243735	-0.1013	0	-0.000185102
**K01966**	0	0	0	-0.159142	-0.047343	0	0
**K01991**	0	0	0	-0.152276	-0.043837	0	0
**K01992**	0	0	0	-0.134762	-0.034409	0	0
**K02004**	0	0	0	-0.070389	0	0	0
**K13652**	0	0	0	-0.210602	-0.080768	0	-3.10E-05
**K02116**	0	0	0	-0.273867	-0.111355	0	-0.000376166
**K09789**	0	0	0	-0.232216	-0.089639	0	-0.000225527
**K02190**	0	0	0	-0.194624	-0.068789	0	-4.64E-06

**K11329**	-0.072135783	0	0	-0.343463	-0.175574	-0.137552739	-0.000678326
**K02503**	0	0	0	-0.16167	-0.047974	0	0
**K11921**	0	0	0	-0.152424	-0.042232	0	0
**K02626**	-0.089433035	0	0	-0.370976	-0.180297	-0.221364456	-0.091849675
**K02651**	-0.01582169	0	0	-0.275012	-0.125556	0	-0.000239933
**K02796**	0	0	0	-0.22771	-0.08302	0	-0.000116722
**K02834**	0	0	0	-0.1676	-0.051744	0	0
**K02871**	0	0	0	-0.170158	-0.052837	0	0
**K02895**	0	0	0	-0.168822	-0.052217	0	0
**K02907**	0	0	0	-0.184729	-0.061573	0	0
**K02931**	0	0	0	-0.169031	-0.052448	0	0
**K02946**	0	0	0	-0.1723	-0.053946	0	0
**K02967**	0	0	0	-0.173326	-0.055016	0	0
**K02996**	0	0	0	-0.171551	-0.053879	0	0
**K03088**	0	0	0	-0.049862	0	0	0
**K07071**	0	0	0	-0.266738	-0.113847	0	-0.000356017
**K03103**	-0.078804859	0	0	-0.352886	-0.164646	-0.163391168	-0.03804852
**K03146**	-0.05682563	0	0	-0.322429	-0.159992	-0.105261657	-0.071587894
**K03154**	0	0	0	-0.190375	-0.064285	0	-1.57E-05
**K08963**	0	0	0	-0.265172	-0.116416	0	-0.000414712
**K03287**	0	0	0	-0.118409	-0.023005	0	0
**K03313**	0	0	0	-0.234379	-0.094214	0	-0.000133201
**K03385**	0	0	0	-0.245474	-0.097669	0	-0.000135205
**K07315**	0	0	0	-0.255686	-0.110748	0	-0.000211376
**K03458**	0	0	0	-0.173315	-0.052126	0	0
**K03517**	0	0	0	-0.177061	-0.056578	0	0
**K03536**	0	0	0	-0.166265	-0.050439	0	0
**K08998**	0	0	0	-0.168722	-0.052089	0	0
**K08737**	-0.04142775	0	0	-0.321156	-0.15464	-0.069886469	-0.000514585
**K03568**	0	0	0	-0.247157	-0.10154	0	-0.000213429
**K11927**	0	0	0	-0.20407	-0.074604	0	-3.09E-05
**K03592**	0	0	0	-0.253284	-0.105985	0	-0.000222556
**K03624**	0	0	0	-0.157403	-0.046482	0	0
**K03719**	0	0	0	-0.178147	-0.057812	0	0
**K05808**	0	0	0	-0.177832	-0.057707	0	0
**K03744**	0	0	0	-0.1833	-0.062132	0	0
**K03779**	0	0	0	-0.271176	-0.1094	0	-0.000394519
**K03801**	0	0	0	-0.227573	-0.087812	0	-0.000105864
**K04047**	0	0	0	-0.241301	-0.101909	0	-0.000195472
**K04618**	-0.008034926	0	0	-0.340232	-0.133061	-0.093699338	0
**K04757**	-0.035473334	0	0	-0.310757	-0.141053	-0.031988902	-0.00052139
**K07358**	0	0	0	-0.367221	-0.157025	-0.214153596	-0.706553974
**K04767**	-0.014931355	0	0	-0.296945	-0.134542	-0.004839685	-0.000416328
**K05340**	0	0	0	-0.240934	-0.099227	0	-0.000189235
**K05367**	-0.004975048	0	0	-0.27663	-0.120041	0	-0.00035134
**K05528**	-0.085525616	0	0	-0.384285	-0.170576	-0.282526219	0
**K05534**	-0.088279137	0	0	-0.367033	-0.189731	-0.192398426	-0.000433024
**K05681**	-0.119277777	0	0	-0.380015	-0.185661	-0.245336452	-0.000663806
**K05841**	-0.078987116	0	0	-0.346595	-0.166896	-0.141395417	-0.000569201
**K05956**	0	0	0	-0.171575	-0.052439	0	0
**K05970**	0	0	0	-0.140653	-0.03706	0	0
**K05989**	0	0	0	-0.134185	-0.033263	0	0
**K06045**	0	0.28689	0	-0.286264	-0.058788	0	-0.000349534
**K12267**	0	0	0	-0.235665	-0.095799	0	-0.000188138
**K06215**	0	0	0	-0.221908	-0.083173	0	-0.000203829
**K07175**	0	0	0	-0.246895	-0.102394	0	-0.000182706
**K06859**	-0.011371616	0	0	-0.285296	-0.129486	0	-0.000289828
**K06896**	0	0	0	-0.150472	-0.043948	0	0

**K06901**	0	0	0	-0.184486	-0.058194	0	0
**K06975**	0	0	0	-0.216455	-0.079801	0	-0.000105001
**K06996**	-0.039797393	0	0	-0.304045	-0.140581	-0.009126519	-0.000415663
**K06999**	0	0	0	-0.261868	-0.109436	0	-0.000242517
**K07001**	0	0	0	-0.161233	-0.048765	0	0
**K07078**	0	0	0	-0.235976	-0.093344	0	-0.00013233
**K07118**	-0.021422825	0	0	-0.297266	-0.135682	0	-0.000319362
**K07165**	0	0	0	-0.091447	-0.008455	0	0
**K07173**	0	0	0	-0.18675	-0.060171	0	-6.98E-05
**K07278**	0	0	0	-0.259482	-0.112107	0	-0.000446557
**K07446**	0	0	0	-0.339391	-0.143146	-0.128062988	-0.943419646
**K07403**	-0.009978521	0	0	-0.288735	-0.133793	0	-0.000449345
**K07488**	-0.013380919	0	0	-0.288027	-0.128846	0	-0.000302027
**K07588**	0	0	0	-0.210234	-0.075886	0	-9.91E-05
**K14979**	-0.068244381	0	0	-0.344536	-0.163859	-0.145617642	-0.000611778
**K07691**	0	0	0	-0.180242	-0.020189	0	-0.234804101
**K07735**	0	0	0	-0.239849	-0.096401	0	-0.000158043
**K08087**	0	0	0.800531	-0.037835	0	0	-0.157263716
**K08222**	-0.064681036	0	0	-0.341549	-0.162816	-0.13017062	-0.052698098
**K08257**	-0.052138788	0	0	-0.310751	-0.149332	-0.034784364	-0.000378413
**K08321**	-0.010641639	0	0	-0.291378	-0.130961	0	-0.000415455
**K08681**	0	0	0	-0.23272	-0.089362	0	-0.0002513
**K09128**	-0.060434302	-0.017596	0	-0.353713	-0.183576	-0.203039951	-0.000431436
**K09704**	0	0	0	-0.201405	-0.07564	0	-2.21E-06
**K09705**	-0.087506732	0	0	-0.362606	-0.18191	-0.224289209	-0.119988193
**K09740**	-0.03104467	0	0	-0.367119	-0.149962	-0.170393657	-0.00045364
**K09758**	0	0	0	-0.245863	-0.099943	0	-0.000150015
**K09793**	0	0	0	-0.261152	-0.115305	0	-0.00023741
**K09859**	0	0	0	-0.330375	-0.140887	-0.059666644	-8.71E-05
**K09888**	0	0	0	-0.182019	-0.059964	0	0
**K09964**	-0.043987457	0	0	-0.378176	-0.152407	-0.230956833	-0.192902467
**K09992**	-0.027825134	0	0	-0.281858	-0.135732	0	-0.000347445
**K10563**	0	0	0	-0.262519	-0.113819	0	-0.000241325
**K10677**	-0.043201402	0	0	-0.32351	-0.148943	-0.079012366	-0.018009234
**K11022**	0	0	0	-0.234973	-0.091893	0	-0.000162759
**K11089**	-0.115211349	0	0	-0.393421	-0.204133	-0.303253608	-0.000339681
**K11294**	0	0	0	-0.214376	-0.081341	0	-1.57E-06
**K11381**	-0.028279006	0	0	-0.285118	-0.131556	0	-0.00027637
**K13444**	-0.056652249	0	0	-0.320278	-0.15735	-0.087394368	-0.000565305
**K12344**	-0.017765544	0	0	-0.287789	-0.132558	0	-0.000231024
**K12599**	0	0	0	-0.325994	-0.134692	-0.075344597	-0.540458799
**K13007**	-0.013339888	0	0	-0.278016	-0.124087	0	-0.000239552
**K13107**	-0.060037086	0	0	-0.378132	-0.191338	-0.237657973	-0.000495749
**K13410**	-0.056869969	0	0	-0.329881	-0.150146	-0.047281171	-3.11E-06
**K14310**	-0.067273514	0	0	-0.397792	-0.170238	-0.265582202	-0.061628887
**K13820**	-0.150586257	-0.273863	0	-0.402114	-0.208283	-0.264772394	0
**K13874**	-0.05745455	0	0	-0.331589	-0.160611	-0.099276567	-0.006296134
**K13993**	0	0	0	-0.152024	-0.042853	0	0
